# On the Origin of the Above-Room-Temperature Magnetism in the 2D van der Waals Ferromagnet Fe_3_GaTe_2_

**DOI:** 10.1021/acs.nanolett.4c01019

**Published:** 2024-06-06

**Authors:** Alberto M. Ruiz, Dorye L. Esteras, Diego López-Alcalá, José J. Baldoví

**Affiliations:** † Instituto de Ciencia Molecular, Universitat de València, Catedrático José Beltrán 2, 46980 Paterna, Spain

**Keywords:** Magnetism, 2D materials, first-principles calculations, spintronics, straintronics

## Abstract

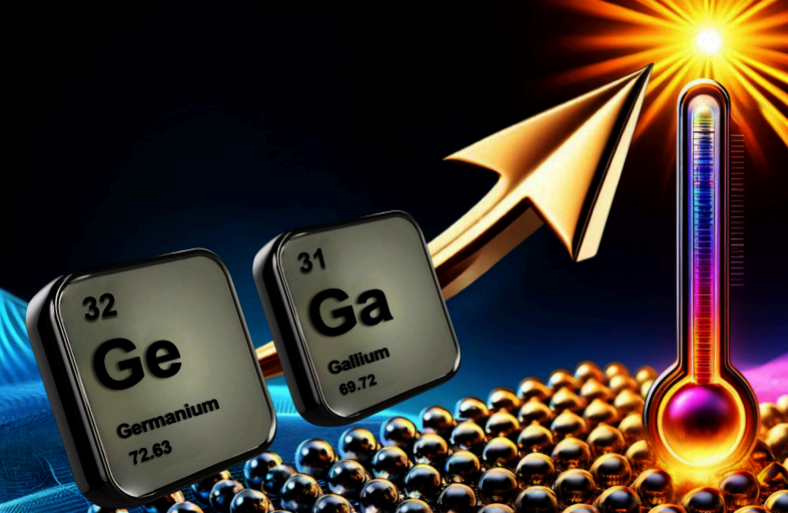

2D magnetic materials have attracted growing interest driven by their unique properties and potential applications. However, the scarcity of systems exhibiting magnetism at room temperature has limited their practical implementation into functional devices. Here we focus on the van der Waals ferromagnet Fe_3_GaTe_2_, which exhibits above-room-temperature magnetism (*T*_c_ = 350–380 K) and strong perpendicular anisotropy. Through first-principles calculations, we examine the magnetic properties of Fe_3_GaTe_2_ and compare them with those of Fe_3_GeTe_2_. Our calculations unveil the microscopic mechanisms governing their magnetic behavior, emphasizing the pivotal role of ferromagnetic in-plane couplings in the stabilization of the elevated *T*_c_ in Fe_3_GaTe_2_. Additionally, we predict the stability, substantial perpendicular anisotropy, and high *T*_c_ of the single-layer Fe_3_GaTe_2_. We also demonstrate the potential of strain engineering and electrostatic doping to modulate its magnetic properties. Our results incentivize the isolation of the monolayer and pave the way for the future optimization of Fe_3_GaTe_2_ in magnetic and spintronic nanodevices.

The recent breakthrough of long-range magnetic order in two-dimensional (2D) van der Waals (vdW) magnetic materials represents an exciting research avenue that opens new pathways for exploring physical phenomena and applications.^1−3^ The discovery of ferromagnetic materials at the 2D limit, such as the semiconductors CrI_3_ and Cr_2_GeTe_6_, has attracted considerable attention since 2017.^1,2^ However, practical applications are drastically hindered by their low critical temperatures below liquid nitrogen temperature.^4,5^ In this regard, materials such as CrSBr or the metallic Fe_3_GeTe_2_ have come to the forefront, retaining magnetic properties down to monolayer thickness with higher Curie temperatures (*T*_c_) of 146 and 130 K, respectively.^6−8^ In this context, external fields, strain engineering, and electrostatic doping have proven to be powerful strategies to improve the magnetic behavior of these systems.^4,9−14^

In particular, Fe_3_GeTe_2_ is a metal distinguished by its resilience after exfoliation^15^ and exhibits a high *T*_c_ with substantial strong magnetic anisotropy,^16,17^ suggesting its potential for spintronics and magnonics applications.^18−22^ While bulk-phase magnetism persists up to 230 K, *T*_c_ is significantly reduced in thinner layers.^6,15^ To address this, several strategies have been employed, such as electron gating,^15^ intercalation,^23−25^ or external pressure.^26^ Among them, increasing the Fe composition in Fe_*x*_GeTe_2_ crystals has proven to be a powerful method to boost the *T*_c_, reaching up to 310 K.^27−29^

More recently, bulk Fe_3_GaTe_2_ has emerged as a magnetic material of interest due to its above-room-temperature magnetism (350–380 K) and strong perpendicular magnetic anisotropy.^30,31^ Its electronic and magnetic properties have been deeply investigated;^32−34^ it has been incorporated into spin-valve, magnetic tunnelling junctions, and proton-intercalated devices^35−40^ and has shown potential for hosting magnetic skyrmions at room temperature.^41−43^ Nevertheless, a comprehensive magnetic analysis to elucidate the main mechanisms governing the high critical temperature of Fe_3_GaTe_2_ and the possibility of maintaining its outstanding magnetic properties at the monolayer limit is still lacking.

In this work, through first-principles calculations, we investigate the fundamental mechanism responsible for room-temperature magnetism in bulk Fe_3_GaTe_2_. Furthermore, we present a comparative analysis with Fe_3_GeTe_2_, unambiguously demonstrating the pivotal role of the competing ferromagnetic-antiferromagnetic in-plane couplings on the critical temperatures of both systems. In addition, we prove the dynamical stability of the Fe_3_GaTe_2_ monolayer and explore the impact of strain engineering and electrostatic doping in the modification of the magnetic and electronic properties of this system.

Fe_3_GaTe_2_ is a van der Waals (vdW) magnetic material, isostructural to the widely known Fe_3_GeTe_2_, that crystallizes within a hexagonal layered structure in the space group *P*63/*mmc* (No. 194). Each unit cell contains two layers of the material stacked along the *c* direction separated by an interlayer spacing of ∼8.1 Å. The lattice parameters are *a* = *b* = 3.986 Å and *c* = 16.229 Å.^30^ Within each layer the system is formed by five atomic sublayers containing two external surfaces of Te atoms, a pair of internal layers formed by two equivalent Fe atoms (Fe_1_ and Fe_2_) and a central layer consisting in Ga and inequivalent Fe_3_ atoms (Figure 1a).

**Figure 1:**
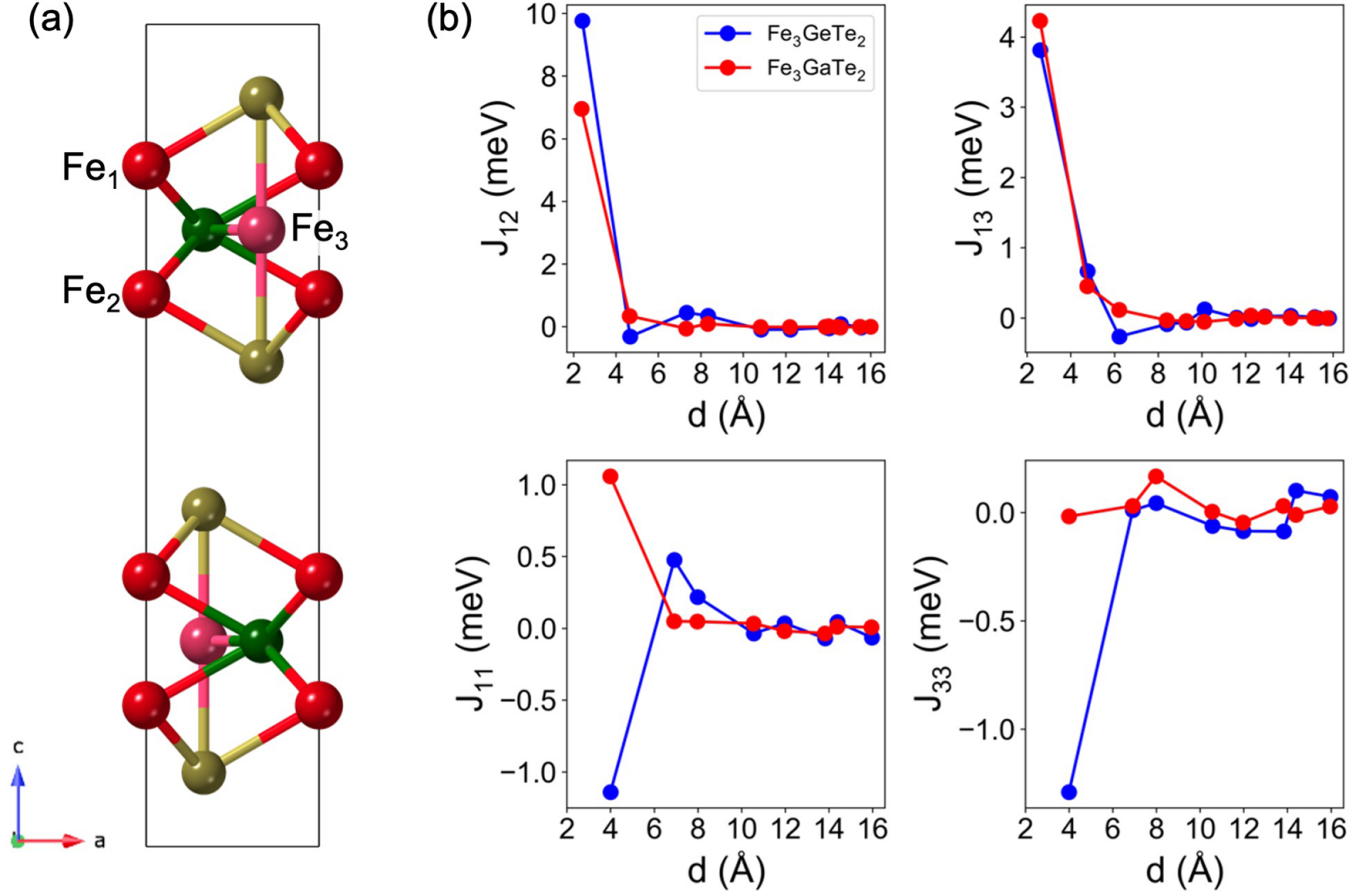
(a) Lateral view of a unit cell of bulk Fe_3_GaTe_2_, which includes two single-layers. Color code: Fe_1,2_ (red), Fe_3_ (pink), Ga (green), and Te (yellow). (b) Interplane exchange interactions *J*_12_, *J*_13_ (top panel) and in-plane couplings *J*_11_ and *J*_33_ (bottom panel) for bulk Fe_3_GaTe_2_ (red) and Fe_3_GeTe_2_ (blue), as well as their evolution with distance to a maximum of 16 Å.

First, we perform first-principles calculations on bulk Fe_3_GaTe_2_ using the LDA scheme. The results reveal magnetic moments of 2.05 and 1.35 μ_B_/Fe for equivalent Fe_1,2_ and inequivalent Fe_3_ atoms, respectively. The calculated average magnetic moment is 1.82 μ_B_/Fe atom, consistent with experimental observations at 3 K and previous theoretical studies.^30,44^ Furthermore, we extracted the magnetic anisotropy energy (MAE) by means of the energy difference between in-plane and out-of-plane magnetic configurations. Our results yield a value of MAE of 0.31 meV/Fe, being in good agreement with previous studies^32,44^ and confirming that the material exhibits a strong perpendicular anisotropy with spins aligned along the *c* axis.

To explore the reasons behind the different *T*_c_ values of Fe_3_GaTe_2_ and Fe_3_GeTe_2_, which are 380 and 230 K, respectively, we determine the magnetic exchange couplings in both materials and perform atomistic simulations (see Methods). The exchange interactions are determined by first formulating a tight-binding Hamiltonian in the basis of maximally localized Wannier Functions (MLWFs)^45^ and then employing the TB2J package,^46^ which is based on the use of Green’s functions. The spin Hamiltonian has the following form:
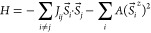
where *J*_*ij*_ represent the isotropic exchange interactions, *S*_*i*_ and *S*_*j*_ are the magnetic moments of the different sites and *A* accounts for the magnetic anisotropy of the system.

Our calculations reveal that the magnetic interaction picture of Fe_3_GaTe_2_ and Fe_3_GeTe_2_ is quite intricate, where emergent global ferromagnetism arises from a competition between different couplings. We categorize these interactions into two groups, namely, (*i*) interplane interactions, which encompass *J*_12_ (interactions between atoms Fe_1_–Fe_2_) and *J*_13_ (Fe_1_–Fe_3_), as well as (*ii*) in-plane exchanges, comprising *J*_11_ (Fe_1_–Fe_1_) and *J*_33_ (Fe_3_–Fe_3_) (Figure 1a). Exchange interactions take place between the Fe species located at different Fe–Fe distances. In constructing the spin Hamiltonian, we have considered the evolution of *J*_12_, *J*_13_, *J*_11_, and *J*_33_ as a function of distance (Figure 1b), between an atom and its atomic neighbor, to a maximum of 16 Å, where they saturate to zero. We also determined the couplings between adjacent layers, which become relatively small compared with interplane and in-plane ones, due to the larger distance between their magnetic centers (see Figure S1 and Tables S1–S4). As presented in Figure 1b, both systems display FM characteristics, with Fe_3_GaTe_2_ exhibiting lower values compared to Fe_3_GeTe_2_ when considering solely the interplane couplings. However, more significant differences appear upon examination of the in-plane exchanges. In Fe_3_GeTe_2_, the *J*_11_ and *J*_33_ interactions are antiferromagnetic (AF), resulting in a geometrically frustrated spin–lattice.^47−49^ Conversely, for Fe_3_GaTe_2_, *J*_11_ and *J*_33_ display values of 1.1 and −0.04 meV, respectively, indicating that the AF contribution of the in-plane couplings is almost suppressed, which, in turn, amplifies the net ferromagnetism. In Supporting Information Section 1.3, we show a comparison of the exchange parameters of Fe_3_GaTe_2_ with those in the existing bibliography, employing a common notation. The *T*_c_ values stand at 641 K for Fe_3_GaTe_2_ and 144 K for Fe_3_GeTe_2_, which deviate from the experimental values of 380 and 230 K, respectively. Nevertheless, our results correctly show the overall trend of a higher critical temperature of Fe_3_GaTe_2_ with respect to that of Fe_3_GeTe_2_. The differences in magnetic couplings between both materials are also studied with GGA functionals and LDA + U (Supporting Information Sections 1.2 and 1.4). While they overall provide comparable results to LDA, the latter triggers AF coupling between layers in Fe_3_GeTe_2_, while the GGA functionals and LDA + U correctly capture an FM ground state. However, both GGA and LDA + U result in underestimated values of the AF J_11_ coupling in Fe_3_GeTe_2_, leading to a pronounced overestimation of its *T*_c_ (reaching 547 K even at *U* = 1 eV and 504 K for GGA functionals). Thus, in subsequent calculations, we employ the LDA functionals, as we focus on the rationalization of the *T*_c_ of both systems.

Considering the chemical and structural similarity between Fe_3_GaTe_2_ and Fe_3_GeTe_2_, which retains ferromagnetism and strong perpendicular anisotropy down to the monolayer limit,^6,15^ we investigate the electronic and magnetic properties of single-layer Fe_3_GaTe_2_. According to our first-principles calculations, the optimized lattice parameters of the monolayer are *a* = *b* = 3.947 Å, being slightly smaller than those of the bulk structure. We observe that a single layer maintains a FM ground state, with magnetic moments of 2.05 and 1.31 μ_B_/Fe for Fe_1,2_ and Fe_3_ atoms, respectively. This yields an average magnetic moment for Fe atoms of 1.80 μ_B_, which is nearly identical to that of the bulk and higher than the calculated value for the Fe_3_GeTe_2_ monolayer (1.61 μ_B_).

Then, we explore the dynamic stability of the Fe_3_GaTe_2_ monolayer by performing phonon calculations. As observed in Figure 2a, the phonon dispersion presents no imaginary frequencies and thus the structure is stable, resembling the one of the Fe_3_GeTe_2_ monolayer.^50^ Furthermore, we study the energetic stability of the Fe_3_GaTe_2_ monolayer by calculating its formation energy relative to the bulk structure. This parameter is defined as the energy difference (per atom) between the monolayer and bulk forms, providing an estimation of the energy required to synthesize a single layer of material from its bulk counterpart. Using this definition, we obtain a value of 36.85 meV/atom, being close to the reported value for the Fe_3_GeTe_2_ monolayer (48.9 meV/atom)^50^ and comparable to results for other vdW materials successfully exfoliated to the monolayer limit.^51^ Regarding the electronic band structure and projected density of states (Figure 2b), one can observe that the system is metallic, with the d orbitals of the transition metal playing an important role around the Fermi level. In contrast, the contributions of Ga and Te are almost negligible, predominantly from the p orbitals of these atoms.

**Figure 2:**
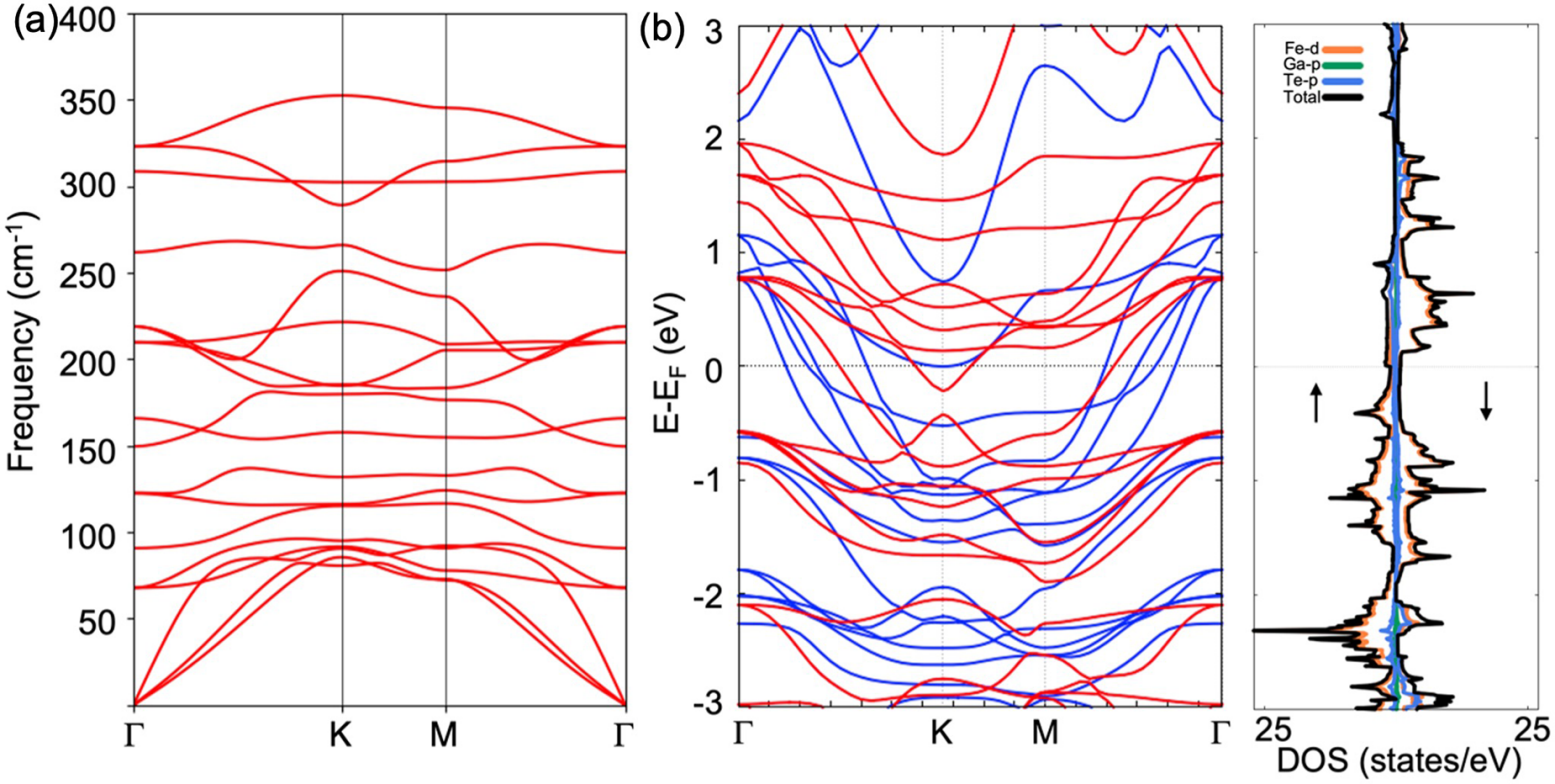
(a) Phonon spectrum and (b) electronic band structure (left) and orbital-resolved density of states (right) of Fe_3_GaTe_2_ monolayer. Blue (red) color in the band structure indicates spin up (down) states.

Furthermore, we estimate the magnetic exchange interactions of the Fe_3_GaTe_2_ monolayer. A direct comparison with the bulk results reveals minor variations, implying that the monolayer is likely to preserve robust FM character (Figure S9). This is further supported by the calculation of the MAE, which yields a value of 0.36 meV/Fe, indicating a substantial perpendicular anisotropy with spins aligned along the *c* axis. Our atomistic simulations predict a high *T*_c_ value of 594 K, which is considerably higher than the one calculated for monolayer Fe_3_GeTe_2_ (100 K).

In Figure 3a we directly compare the exchange interactions in monolayers Fe_3_GaTe_2_ and Fe_3_GeTe_2_. Our analysis indicates that, as in bulk, the in-plane couplings *J*_11_ and *J*_33_ are the origin of the observed differences in *T*_c_ between these compounds. The distinct contributions originate from specific orbitals involved in the stabilization of long-range magnetic ordering in each system, as confirmed by our orbital-resolved analysis (Figure 3b,c). The ferromagnetic *J*_12_ interaction is predominantly governed by d_*z*^2^_–d_*z*^2^_, d_*xz*_–d_*xz*_, and d_*yz*_–d_*yz*_ orbitals, with a small antiferromagnetic contribution arising from in-plane d_*xy*_–d_*xy*_ and d_*x*^2^–*y*^2^_–d_*x*^2^–*y*^2^_. The overall reduction in ferromagnetism within *J*_12_ for Fe_3_GaTe_2_ can be attributed to a diminished FM contribution from d_*xz*_–d_*xz*_ and d_*yz*_–d_*yz*_ orbitals compared to that of Fe_3_GeTe_2_ (Figure S10). This orbital-resolved description contrasts with the findings of Lee et al.,^32^ who associated the increase of *T*_c_ in Fe_3_GaTe_2_ with a higher value of *J*_12_ relative to Fe_3_GeTe_2_. Additionally, the FM nature of *J*_11_ is attributed to a predominant FM d_*yz*_–d_*xz*_ superexchange pathway mediated by the p_*y*_ and p_*x*_ orbitals of Ga and Te, respectively. This mechanism is highly diminished in Fe_3_GeTe_2_, eventually turning to become AF (Figure 3b). In addition, the *J*_33_ in Fe_3_GeTe_2_ is primarily sourced from an AF d_*z*^2^_–p_*x*_–d_*z*^2^_ mechanism mediated by Ge, which is nearly suppressed in Fe_3_GaTe_2_ (Figure 3c). We discard the possibility that structural differences between both systems are the determinant factor to explain the variations in magnetic exchange couplings and the differences in *T*_c_ (Figure S11).

**Figure 3:**
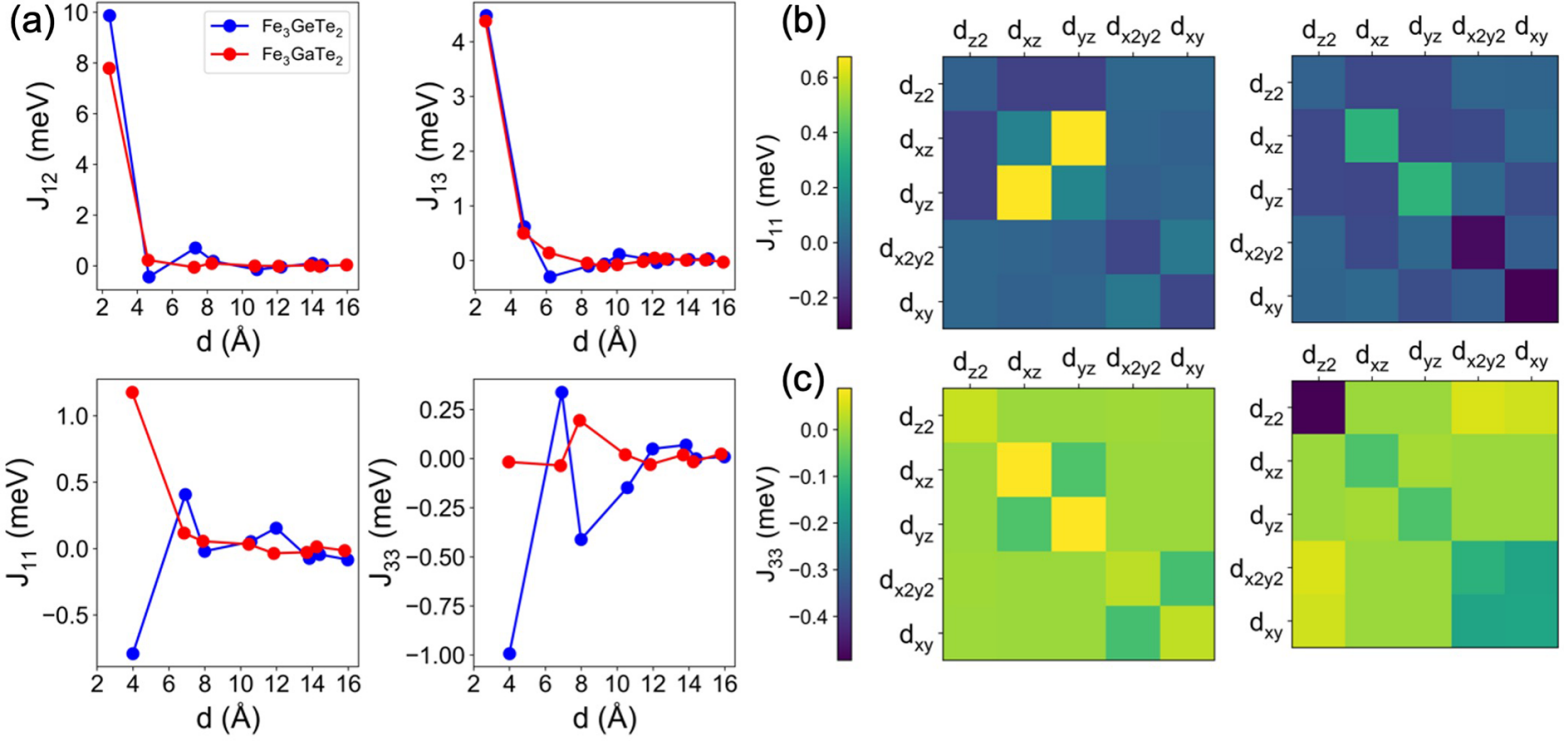
(a) Interplane exchange interactions *J*_12_ and *J*_13_ (top panel) and in-plane couplings *J*_11_ and *J*_13_ (bottom panel) for monolayers Fe_3_GaTe_2_ (red) and Fe_3_GeTe_2_ (blue) along with their evolution with distance to a maximum of 16 Å. Orbital-resolved in-plane exchange parameters *J*_11_ (b) and *J*_33_ (c) for Fe_3_GaTe_2_ (left) and Fe_3_GeTe_2_ (right) monolayers.

To assess the impact of *J*_11_ and *J*_33_ on the FM stabilization, we carry out calculations considering interactions until 16 Å (Figure S12). By solely including interactions up to 3 Å (accounting only for *J*_12_ and *J*_13_), we obtain values of *T*_c_ of 251 K for Fe_3_GaTe_2_ and 220 K for Fe_3_GeTe_2_. This slight variation in *T*_c_ between both systems suggests that a model restricted to nearest-neighbor interplane couplings is not adequate to describe the magnetic behavior. Extending to 4 Å, where *J*_11_ and *J*_33_ are also considered, the *T*_c_ of Fe_3_GaTe_2_ is enhanced up to 434 K, whereas the *T*_c_ of Fe_3_GeTe_2_ is drastically reduced to 0 K. This proves the key role of in-plane couplings and the crucial significance of the AF character of those in the lower *T*_c_ of Fe_3_GeTe_2_. We remark the relevant effects of *J*_11_ and *J*_33_ that contribute to *T*_c_ with a larger number of interactions (6 nearest-neighbors each), balancing the effect of the higher magnitudes of *J*_12_ and *J*_13_, that contribute solely with 1 and 3 neighbors, respectively (see Tables S8 and S9). Finally, expanding interactions to 16 Å provides *T*_c_ values of 594 and 100 K, highlighting the importance of long-range magnetic couplings. Furthermore, *T*_c_ changes slightly with values of 0 < MAE < 2 meV, increasing steadily for MAE > 2 meV and reaching an enhancement of 50 K upon a value of MAE of 8 meV (Figure S13).

Additionally, we investigate the evolution of the magnetic properties of the Fe_3_GaTe_2_ monolayer upon mechanical deformation and electrostatic doping. Figure 4a–c illustrates the variation of the magnetic moments for both Fe and ligands, along with the dependence of MAE and exchange parameters under biaxial strain. Notably, under tensile strain, the average Fe magnetic moments steadily increase, remaining almost null in the ligands. The MAE results reveal that the magnetization easy axis remains off-plane in the entire range. Its value is almost null at −4% and reaches a maximum at 1%, decreasing at values of ε > 1%. According to our simulations, compression values slightly exceeding ε = −4% would induce in-plane magnetization within the system. On the other hand, a more substantial tensile strain would be required to modify the off-plane magnetization easy axis. Besides the evolution of MAE reflecting the trend observed in the magnetic moments of Fe_3_, the change in the latter is considerably smaller than the variation in MAE. This suggests that the drastic change in MAE is not primarily due to spin moment variations. Prior studies have attributed this phenomenon to band shifts involving significant spin–orbit coupling.^52^ This is supported by our band structure calculations upon applied strain, demonstrating effective modulation of the electronic properties around the Fermi level (Figure S15). The evolution of the MAE diverges from that reported for Fe_3_GeTe_2_, where it parallels the evolution of magnetic moments and increases continuously upon tensile strain.^50^ In addition, analyzing the magnetic couplings, Figure 4c shows that the FM exchange couplings exhibit an increase (decrease) upon compression (elongation) over the studied range. Notably, at ε < −2%, *J*_12_ significantly increases while *J*_11_ decreases, the latter transitioning toward an AF state. We attribute these anomalous evolutions to the different trend of magnetic moments for ε < −2% compared to the rest of the map. Note that Fe_1_ and Fe_2_ atoms are situated within the same *xy* plane and consequently the distance Fe_1_–Fe_2_ remains intact upon applied strain. However, the angle governing the Fe_1_–Ga–Fe_2_ superexchange pathway is reduced from 57.1° (−4%) to 53.3° (+4%) upon tensile strain, thereby having an impact in the coupling *J*_12_ (Figure S14). We computed the *T*_c_ at different levels of strain (Figure 4d), observing that besides *J*_12_, *J*_13_ and *J*_33_ are enhanced with respect to the undistorted structure at ε = −4%. The rapid decrease of *J*_11_ results in a drop of the *T*_c_ to a value of 320 K; conversely, a 4% elongation yields a *T*_c_ reduction by 10%.

**Figure 4:**
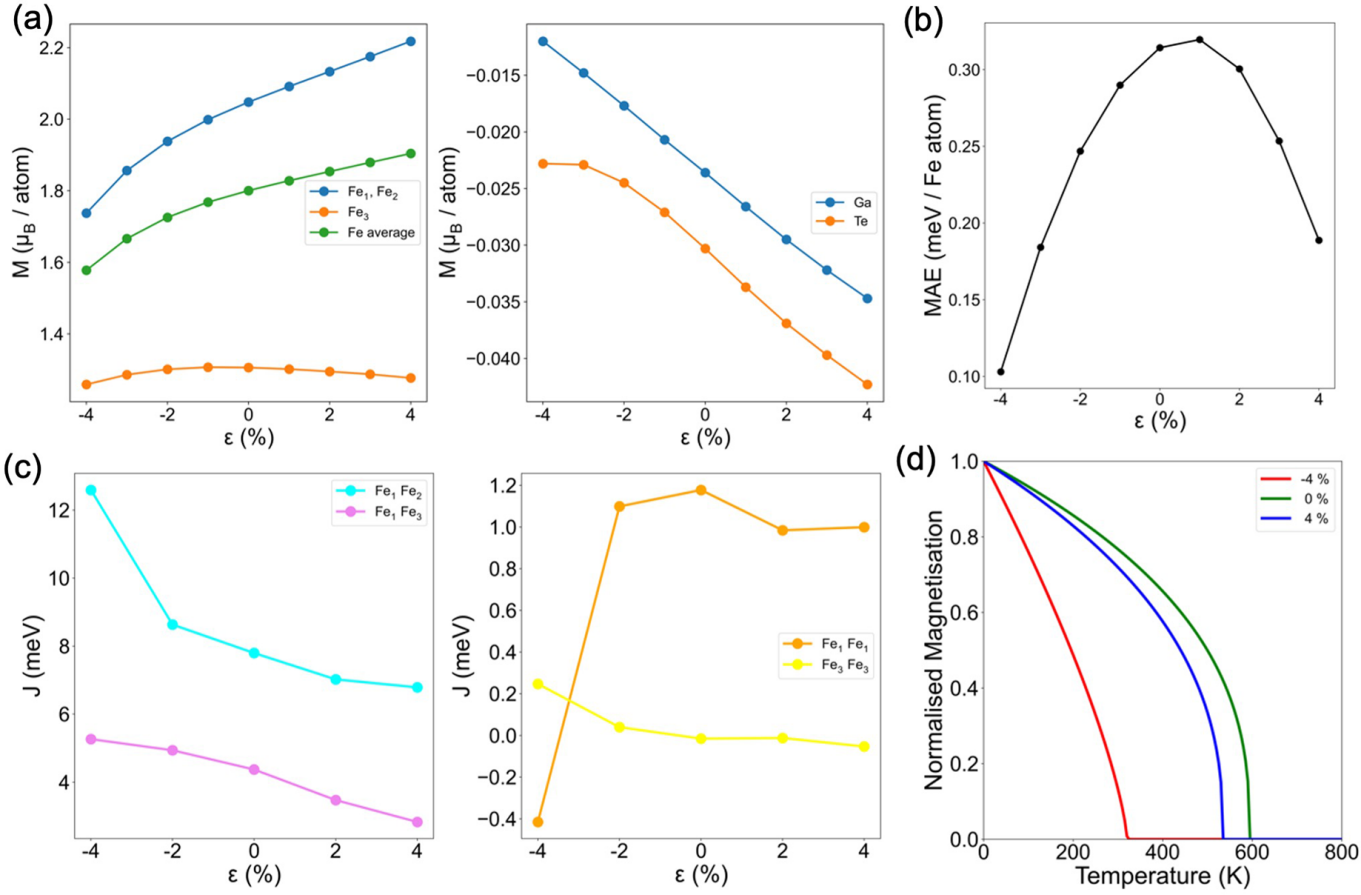
Evolution of (a) magnetic moments of metals and ligands (left and right panels, respectively), (b) MAE, (c) inter- (left) and in-plane (right) exchange parameters, and (d) *T*_c_ of Fe_3_GaTe_2_ monolayer upon applied strain.

The analysis of the impact of electrostatic doping on the magnetic properties shows that the magnetic moments of both equivalent and inequivalent Fe atoms are similarly affected, increasing (decreasing) under electron (hole) doping (Figure 5a). In contrast, the magnetic moments of Ga remain almost unchanged, whereas those of Te experience a pronounced increase under electron doping. Regarding the evolution of MAE reported in Figure 5b, one can observe that the magnetization easy axis changes from off-plane to in-plane with a hole carrier density of 1.1 × 10^14^ cm^–2^. This agrees well with previous theoretical observations of in-plane magnetism in bulk Fe_3_GaTe_2_ upon hole doping.^32^ The change in MAE agrees well with an effective shift of the bands around the high symmetry points Γ and K (Figure S18), which are postulated to significantly influence the anisotropy of Fe_3_GeTe_2_ and Fe_3_GaTe_2_.^32,52^ We note the presence of hole (around Γ) and electron (around K) pockets, which are shifted upward (downward) upon hole (electron) doping. Analyzing the evolution of magnetic couplings, we observe that electron doping leads to an increase of the FM interactions *J*_12_ and *J*_13_, suggesting a potential rise in *T*_c_ (Figure 5c). However, there is a softening of the FM character of *J*_11_ and *J*_33_ that results in a reduced *T*_c_ of 501 K (Figure 5d). This contrasts with the increase of the critical temperature reported for Fe_3_GeTe_2_ upon electron gating.^15^ On the other hand, hole doping leads to a slight reduction in *J*_12_ and *J*_13_, while *J*_11_ and *J*_33_ become more FM, resulting in an almost unchanged critical temperature. This aligns with recent experimental findings in bulk Fe_2.84_GaTe_2_, where Fe deficiency is linked to a slightly lower *T*_c_ with respect to Fe_3_GaTe_2_.^41^

**Figure 5:**
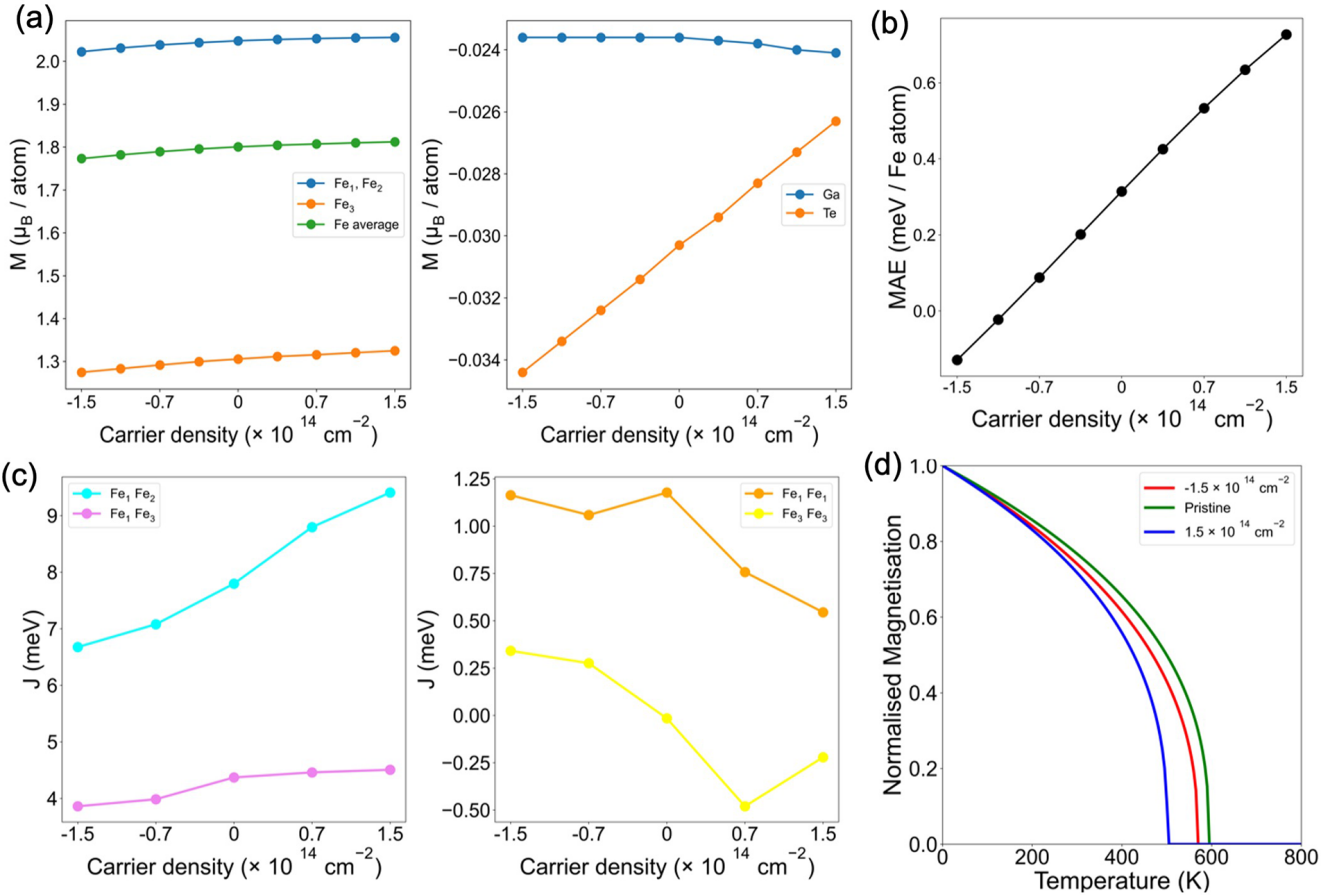
Evolution upon hole and electron doping of (a) magnetic moments of Fe, Ga, and Te (left and right panels, respectively), (b) MAE, (c) inter- (left) and in-plane (right) exchange parameters, and (d) *T*_c_ of Fe_3_GaTe_2_ monolayer. A positive (negative) sign of the carrier density corresponds to an excess of electrons (holes).

In summary, we investigate the origin of the above room-temperature magnetism of Fe_3_GaTe_2_ via first-principles calculations. We conduct a thorough comparison of its magnetic properties with its isostructural counterpart Fe_3_GeTe_2_, unambiguously demonstrating that the AF/FM character of their intralayer *J*_11_ and *J*_33_ couplings is the determinant factor for the higher *T*_c_ in Fe_3_GaTe_2_. We provide an orbital-resolved analysis of their magnetic exchange interactions showing the microscopic mechanisms governing the enhanced *T*_c_ of Fe_3_GaTe_2_. This study is extended to the monolayer limit, where we demonstrate the dynamical stability of Fe_3_GaTe_2_ by means of phonon calculations, as well as the presence of strong perpendicular anisotropy and elevated *T*_c_. Finally, the tunability of the magnetic properties of the Fe_3_GaTe_2_ monolayer is proven through strain engineering and electrostatic doping, illustrating the effectiveness of these techniques to manipulate its complex magnetic picture as well as their potential to tune the anisotropy of the system. Our findings lay a foundation for comprehending the origin of the critical temperature *T*_c_ in both Fe_3_GaTe_2_ and Fe_3_GeTe_2_ and their future manipulation in magnetic and spintronic devices.

## Methods

We carried out spin-polarized density-functional theory (DFT) using the Quantum ESPRESSO package.^53^ Spin-polarized local density approximation (LDA)^54^ was used to approximate the exchange–correlation functional given that it has been proved to properly describe properties of Fe_3_GaTe_2_ and Fe_3_GeTe_2_,^15,44,55^ avoiding an overestimation of magnetic moments.^50^ We also analyzed the magnetic properties of both materials employing GGA functionals and LDA + U (see Sections 1.2 and 1.4 of the Supporting Information, respectively). For the bulk structures we relaxed the atomic coordinates while for the monolayers both atomic coordinates and lattice parameters were optimized. In both cases, the optimizations were carried out until forces on each atom were smaller than 10^–3^ Ry/au and the energy difference between two consecutive relaxation steps was less than 10^–4^ Ry. The electronic wave functions were expanded with well-converged kinetic energy cutoffs for the wave functions (charge density) of 75 (850) Ry for the LDA functionals and 90 (700) Ry for GGA. When using the GGA functionals, Grimme-D3 dispersion corrections were added to account for van der Waals interactions between layers. To properly describe the monolayers, a vacuum spacing of 18 Å was set along the *c* direction to avoid unphysical interactions between layers. For the bulk (monolayer) structures, the Brillouin zone was sampled by a fine Γ-centered 10 × 10 × 3 (10 × 10 × 1) k-point Monkhorst–Pack mesh, that was expanded to 15 × 15 × 3 (15 × 15 × 1) for the calculations of MAE. The phonon spectrum was computed using a 3 × 3 × 1 supercell using the Phonopy code.^56^ A tight-binding model based was constructed based on the maximally localized Wannier functions as implemented in the Wannier90 code.^57^ Our reduced basis set is formed by the d orbitals of Fe and p orbitals of Ga, Ge, and Te. Magnetic exchange interactions were determined using Green’s function method as implemented in TB2J code^46^ employing a 30 × 30 × 5 (30 × 30 × 1) supercell for the bulk (monolayer) structures. The *T*_c_ was obtained by performing atomistic simulations as implemented in the VAMPIRE code^58^ using 10000 equilibration steps and 10000 averaging steps in our calculations employing the llg-heun integrator. Furthermore, we selected supercells of dimensions 40 × 40 × 40 (40 × 40 × 1) for bulk (monolayer) calculations.

To validate the obtained magnetic exchange couplings determined by the plane wave method, we doublechecked our results using a localized atomic orbital approach as implemented in the SIESTA package^59^ (Figures S21 and S22), in which the local density approximation (LDA) was used to describe the exchange–correlation energy.^60,61^ We used a double-ζ basis set for all atoms, and core electrons were described using norm-conserving Troullier–Martins pseudopotentials. A real-space mesh cutoff of 500 Ry and a 64 × 64 × 10 (64 × 64 × 1) Monkhorst–Pack k-point mesh was used for the bulk (monolayer) calculations.

## Supporting Information Available

Magnetic exchange coupling and *T*_c_ with supporting figures and tables; strain engineering of Fe_3_GaTe_2_ monolayer with supporting figures; electrostatic doping Fe_3_GaTe_2_ monolayer with supporting figures; figures of SIESTA calculations (PDF)
