# Supplementary material for: On the Origin of the Above-Room-Temperature Magnetism in the 2D van der Waals Ferromagnet Fe3GaTe2
Source: Nano Lett. 2024 Jun 6;24(26):7886–94. doi: 10.1021/acs.nanolett.4c01019 (PMC11229069; doi:10.1021/acs.nanolett.4c01019)
Supplement: Supplementary file 1 — nl4c01019_si_001.pdf [file nl4c01019_si_001.pdf]

Supporting Information

On the Origin of the Above-Room-Temperature Magnetism in the 2D van der Waals Ferromagnet Fe_3GaTe_2

Authors: Alberto M. Ruiz[†], Dorye L. Esteras[†], Diego López-Alcalá[†] and José J. Baldoví[†,*]

[†]Instituto de Ciencia Molecular, Universitat de València, Catedrático José Beltrán 2, 46980 Paterna, Spain. E-mail: j.jaime.baldovi@uv.es

Table of Contents

1. **Magnetic exchange couplings and T_c 2**
   - 1.1 **Intralayer and interlayer exchange couplings for bulk Fe_3GaTe_2 and Fe_3GeTe_2** 2
   - 1.2 **Comparison between LDA and GGA functionals** 4
   - 1.3 **Comparison of exchange couplings and T_c with existing bibliography** 7
   - 1.4 **Comparison between LDA and LDA + U** 8
   - 1.5 **Comparison between bulk and monolayer Fe_3GaTe_2** 11
   - 1.6 **Monolayer Fe_3GaTe_2** 12
2. **Strain engineering of Fe_3GaTe_2 monolayer 15**
3. **Electrostatic doping Fe_3GaTe_2 monolayer 17**
4. **SIESTA calculations 19**

1. Magnetic exchange couplings and T_c

1.1 Intralayer and interlayer exchange couplings for bulk Fe_3GaTe_2 and Fe_3GeTe_2

To rationalize the differences in the interlayer couplings between Fe_3GaTe_2 and Fe_3GeTe_2 as well as their crossover with the intralayer ones, we extracted the magnetic couplings between different layers (J_{15}, J_{24}, J_{16}, J_{26}, J_{34}, J_{35}, J_{14}, J_{25} and J_{36}). As we observe in Tables S2 and S4, we find the interactions between Fe_2-Fe_4 (J_{24}) are the most significative interactions (due to their shortest distance) with values of 0.154 meV in Fe_3GaTe_2 and -0.364 meV in Fe_3GeTe_2. Nevertheless, they result to be relatively small once compared with the intralayer interactions (Tables S1 and S3).

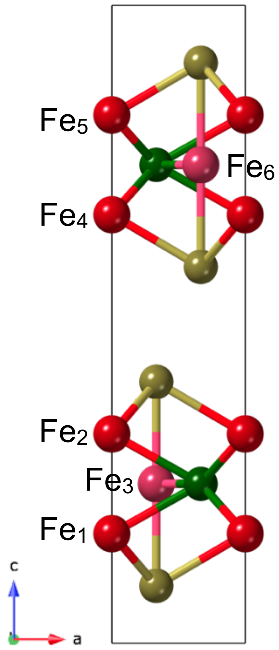

Figure S1. Lateral view of a unit cell of bulk Fe_3GaTe_2, which includes two single-layers. Colour code: $Fe_{1,2}$ (red), Fe_3 (pink), Ga, Ge (green) and Te (yellow).

Table S1. Values of intralayer exchange interactions J_{12}, J_{13}, J_{11} and J_{33} for Fe_3GaTe_2 bulk, along with the number of nearest neighbours (NN) and the distances between them (d). For the comparison, we include the interactions J_{23} and J_{22}, given that they are equivalent to J_{13} and J_{11}, respectively.

Interaction	Value (meV)	NN	d (Å)
J_{12}	6.96	1	2.37
J_{13} (J_{23})	4.23	3 (3)	2.59
J_{11} (J_{22})	1.06	6 (6)	3.99
J_{33}	-0.02	6	3.99

Table S2. Interlayer exchange interactions for Fe_3GaTe_2 bulk along with the number of nearest neighbours (NN) and the distance between them (d).

Interaction	Value (meV)	NN	d (Å)
J_{15}	0.154	1	5.744
J_{24}	0.154	1	5.744
J_{16}	0.072	3	7.301
J_{26}	0.072	3	7.301
J_{34}	0.072	3	7.301
J_{35}	0.072	3	7.301
J_{14}	-0.001	2	8.114
J_{25}	-0.001	2	8.114
J_{36}	-0.092	6	8.435

Table S3. Values of intralayer exchange interactions J_{12}, J_{13}, J_{11} and J_{33} for Fe_3GeTe_2 bulk, along with the number of nearest neighbours (NN) and the distances between them (d). For the comparison, we include the interactions J_{23} and J_{22}, given that they equivalent to J_{13} and J_{11}, respectively.

Interaction	Value (meV)	NN	d (Å)
J_{12}	9.77	1	2.41
J_{13} (J_{23})	3.82	3 (3)	2.60
J_{11} (J_{22})	-1.17	6 (6)	3.99
J_{33}	-1.28	6	3.99

Table S4. Values of interlayer exchange interactions for bulk Fe_3GeTe_2 along with the number of nearest neighbours (NN) and the distance between them (d).

Interaction	Value (meV)	NN	d (Å)
J_{15}	-0.364	1	5.756
J_{24}	-0.361	1	5.756
J_{16}	0.156	3	7.333
J_{26}	0.157	3	7.333
J_{34}	0.155	3	7.333
J_{35}	0.151	3	7.333
J_{14}	-0.144	2	8.168
J_{25}	-0.144	2	8.168
J_{36}	0.073	6	8.487

1.2 Comparison between LDA and GGA functionals

In this section we discuss the effect of the functional employed on the magnetic properties of bulk Fe_3GaTe_2 and Fe_3GeTe_2.

Table S5. Calculated magnetic moments for equivalent $Fe_{1,2}$, inequivalent Fe_3 and averaged (Avg) magnetic moments for bulk Fe_3GaTe_2 and Fe_3GeTe_2 using LDA and GGA functionals.

	μ_B ($Fe_{1,2}$)	μ_B (Fe_3)	μ_B (Avg)
Fe_3GaTe_2			
LDA	2.05	1.35	1.82
GGA	2.28	1.53	2.03
Fe_3GeTe_2			
LDA	2.01	1.13	1.72
GGA	2.44	1.63	2.17

Analysing Table S5, we observe that the calculated average magnetic moments are 1.82 and 1.72 μ_B/Fe atom for Fe_3GaTe_2 and Fe_3GeTe_2, respectively, using the LDA functional, agreeing well with experimental findings[1–4]. On the other hand, GGA functionals tend to overestimate the magnetic moments of both systems, as previously reported[5,6].

However, for reassessing that the main conclusions of our work do not change with the functional employed, in Figure S2 we show a direct comparison of the exchange parameters J_{12}, J_{13}, J_{11} and J_{33} between Fe_3GaTe_2 and Fe_3GeTe_2 employing the GGA functional. From the results of Figure S2 we extract that the interaction picture is the same as in the LDA approach (Figure S3 and Figure 1b), showing that the AF couplings J_{11} and J_{33} are the main driven force for the lower T_C of Fe_3GeTe_2 with respect Fe_3GaTe_2.

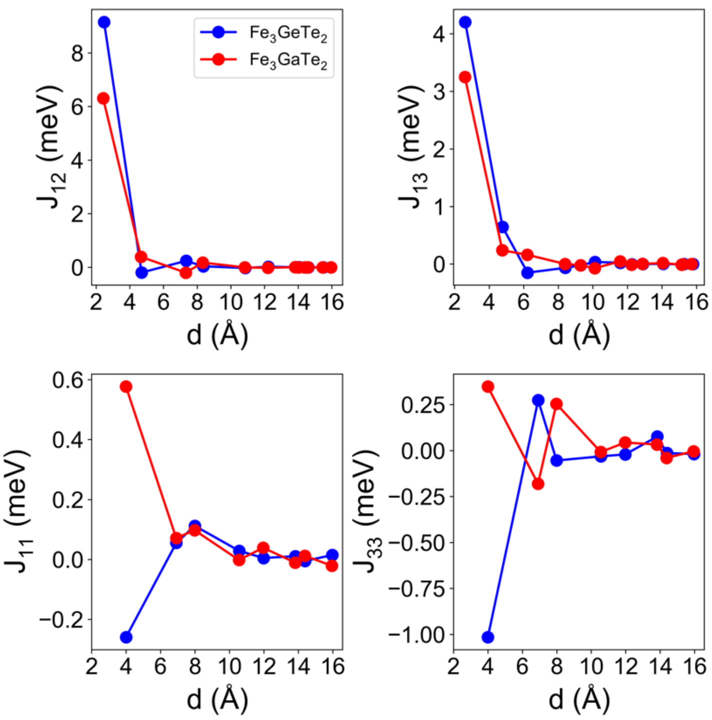

Figure S2. Inter-plane exchange interactions J_{12}, J_{13} (top panel) and in-plane couplings J_{11} and J_{33} (bottom panel) for bulk Fe_3GaTe_2 (red) and Fe_3GeTe_2 (blue), as well as their evolution with distance to a maximum of 16 Å using GGA functionals.

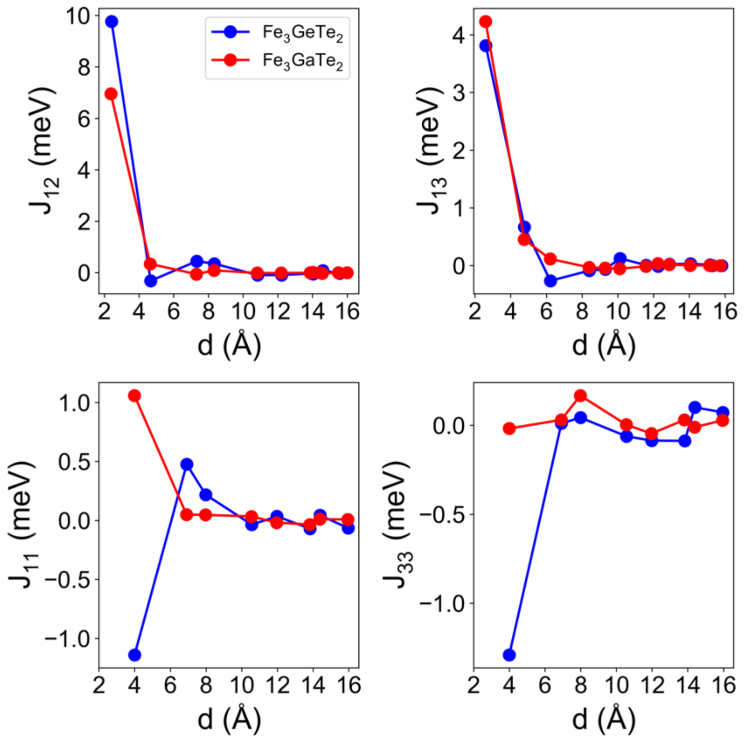

Figure S3. Inter-plane exchange interactions J_{12}, J_{13} (top panel) and in-plane couplings J_{11} and J_{33} (bottom panel) for bulk Fe_3GaTe_2 (red) and Fe_3GeTe_2 (blue), as well as their evolution with distance to a maximum of 16 Å using LDA functionals.

From the results presented in Figures S2 and S3 we observe that the tendencies in the variations in the AF-FM in-plane exchange interactions between Fe_3GaTe_2 and Fe_3GeTe_2 remains intact independently of the functional employed. The values of T_C derived from the calculated exchange couplings at the GGA level are 594 K for Fe_3GaTe_2 and 504 K for Fe_3GeTe_2. The value of T_C of Fe_3GaTe_2 is lower once compared with the LDA scheme (641K in the latter), however there is a large overestimation of the T_C of Fe_3GeTe_2 (which is 144K employing LDA functionals). The overestimated T_C of Fe_3GeTe_2 with GGA functionals arises from the fact that J_{11} = -0.26 meV, compared with the results of J_{11} = -1.17 meV extracted from the LDA calculations. Comparing with previous reports[7], bulk Fe_3GeTe_2 show comparable values of J_{11} and J_{33}, that are better described with the LDA scheme.

Furthermore, we also tested the influence of the functional employed in the magnetic interlayer ground state. For such purpose, we computed the energy difference between the AF and FM magnetic states (ΔE) for Fe_3GaTe_2 and Fe_3GeTe_2 for both functionals (Table S6).

Table S6. Calculated energy difference (ΔE) per Fe atom for bulk Fe_3GaTe_2 and Fe3GeTe2 using LDA and GGA functionals. ΔE refers to the energy difference between the AF and FM interlayer couplings ($\Delta E = E_{AF}-E_{FM}$).

	Fe_3GaTe_2	Fe_3GeTe_2
Functional	ΔE (meV/Fe)	ΔE (meV/Fe)
LDA	2.83	-3.21
GGA	4.09	1.27

From Table S6 we extract that for Fe_3GaTe_2 the interlayer ground state is FM regardless of the functional employed. On the other hand, for Fe_3GeTe_2 the LDA functional results in an AF ground state, while the GGA scheme correctly captures the FM interlayer coupling. This discrepancy has been addressed in prior studies[8], showing that the experimentally observed FM state is achieved upon hole doping. The reported level of hole doping necessary to obtain a FM ground state closely corresponds to the Fe defect concentration observed in experiments (where $Fe_{3-x}GeTe_2$ is likely to be formed)[2,3,9].

1.3 Comparison of exchange couplings and T_c with existing bibliography

Table S7. Comparison of the exchange couplings (in units of meV) between our work at the LDA and GGA level and the reported from Li *et al.*[6] and Lee *et al.*[10]

Interaction	This work (LDA)	This work (GGA)	Li *et al.*[6] (LDA)	Lee *et al.*[10] (GGA)	d (Å)
J_{12}	30.72	35.52	57.18	74.83	2.37
J_{13}	12.19	10.42	17.02	16.90	2.59
J_{11}	4.68	3.25	-0.92	2.866	3.99
J_{33}	-0.04	0.64	2.66	-0.73	3.99
J_{12}	1.53	2.18	-1.55	0.58	4.64
J_{13}	1.30	0.77	-1.29	2.65	4.75
J_{24}	0.68	0.29	3.43	0.37	5.74
T_c (6 Å)	543	451	330	600	-

Table S7 summarizes a direct comparison between our exchange parameters with the existing bibliography in a common reference, using the following Hamiltonian:

$$H = -\sum_{i \neq j} J_{ij} \vec{S}_i \cdot \vec{S}_j$$

where J_{ij} represent the isotropic exchange interactions, S_i and S_j are normalized to 1. Note that as the spin information is included inside J_{ij}, some discrepancies in the exchange parameters can originated from the different magnetic moments of Fe atoms among the different works.

Upon closer examination of the calculations by Li *et al.*[6] and Lee *et al.*[10], we find some remarkable aspects. Firstly, it is noteworthy that there are discrepancies in the magnitude of J_{12} among the different works, with our calculations showing the smallest values for J_{12}. Nevertheless, our results demonstrate an overall notable consistency with the magnetic couplings reported by Lee *et al.*[10]. While in our work (both at the LDA and GGA level) and Lee *et al.*[10] demonstrate a FM coupling for J_{11}, Li *et al.*[6] obtain an AF coupling for it (-0.92). In addition, there is also a discrepancy in the FM or AF character of J_{12} and J_{13} (at distances of 4.64 and 4.75 Å, respectively). These exchange couplings are reported as AF in Li *et al.*[6], while our simulations (both at LDA and GGA level) and those reported by Lee *et al.*[10] indicate a FM behaviour.

Furthermore, by utilizing the reported exchange parameters from publications and summarized in Table S7 (up to a maximum exchange range of 6Å), we conducted calculations of the Curie temperature, thus facilitating a direct comparison across various studies. From the results, we

observe a robust agreement between the T_C derived from our magnetic couplings and those of Lee *et al.*[10]. Additionally, it is also compatible with the one of Li *et al.*[6] considering the challenging nature of atomistic simulations in the determination of T_C.

1.4 Comparison between LDA and LDA + U

To assess for the impact of Hubbard U in the magnetic features of both compounds, we performed a screening of these properties varying the value of U from 0 to 3 eV. In Figure S4, we observe that the LDA + U results in overestimated magnetic moments, while they are correctly captured employing LDA.

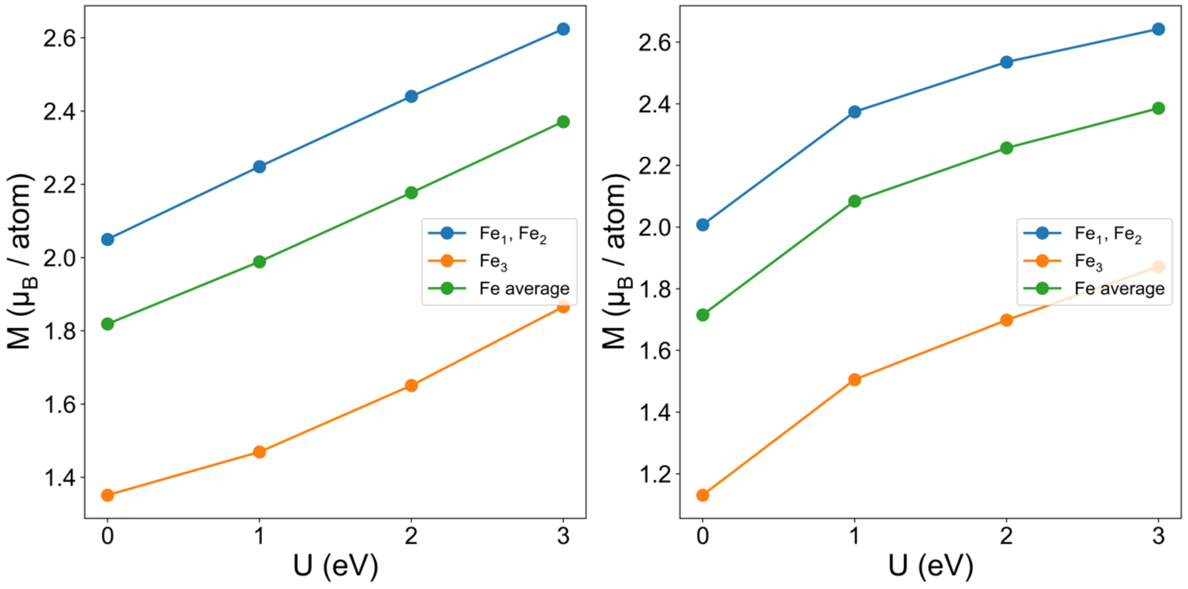

Figure S4. Evolution of magnetic moments of equivalent Fe_1, Fe_2 atoms, inequivalent Fe_3 atoms as well their average value for Fe_3GaTe_2 (left) and Fe_3GeTe_2 with varying Hubbard U from 0 to 3 eV.

We further evaluated the evolution of the exchange parameters J_{12}, J_{13}, J_{11} and J_{33} for both compounds upon the addition of U as well as the resulted T_C (Figures S5-7). From the results of the exchange parameters, we observe that the addition of U in Fe_3GaTe_2 results in an enhancement of J_{12}, which would result in a larger T_C. However, the FM interaction J_{11} is hardly suppressed, being almost null at U = 3 eV. This results in an overall constant T_C in the range of study. On the other hand, the inclusion of U in Fe_3GeTe_2 triggers a drastic reduction of the AF coupling J_{11} even at U=1eV, even turning to become FM for values of U = 2 and 3 eV. This results in highly overestimated T_c as shown in Figure S7.

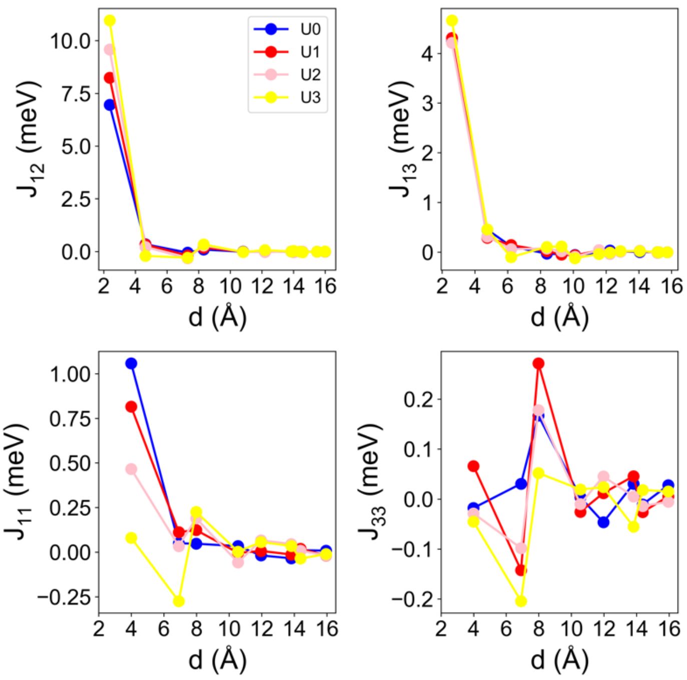

Figure S5. Inter-plane exchange interactions J_{12}, J_{13} (top panel) and in-plane couplings J_{11} and J_{33} (bottom panel) for bulk Fe_3GaTe_2, as well as their evolution with distance upon varying the value of U from 0 to 3 eV.

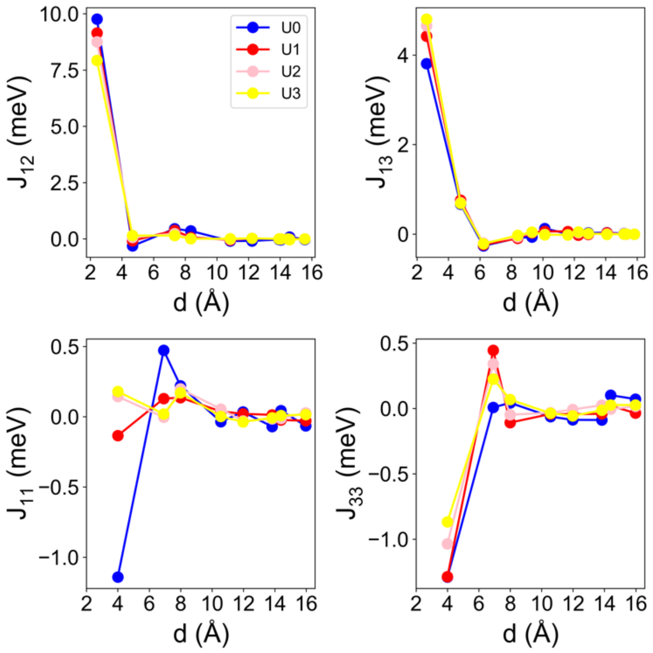

Figure S6. Inter-plane exchange interactions J_{12}, J_{13} (top panel) and in-plane couplings J_{11} and J_{33} (bottom panel) for bulk Fe_3GeTe_2, as well as their evolution with distance upon varying the value of U from 0 to 3 eV.

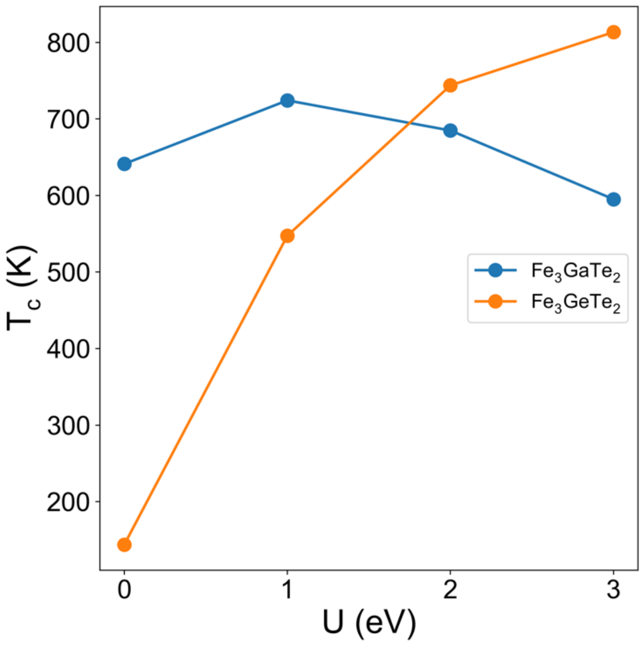

Figure S7. Evolution of the T_C for Fe_3GaTe_2 and Fe_3GeTe_2 as a function of the value of U.

We also tested the influence of the addition of Hubbard U in the magnetic interlayer ground state. For such purpose, we computed the energy difference between the AF and FM magnetic states (E_{AF}-E_{FM}) for Fe_3GaTe_2 and Fe_3GeTe_2 varying U (Figure S8).

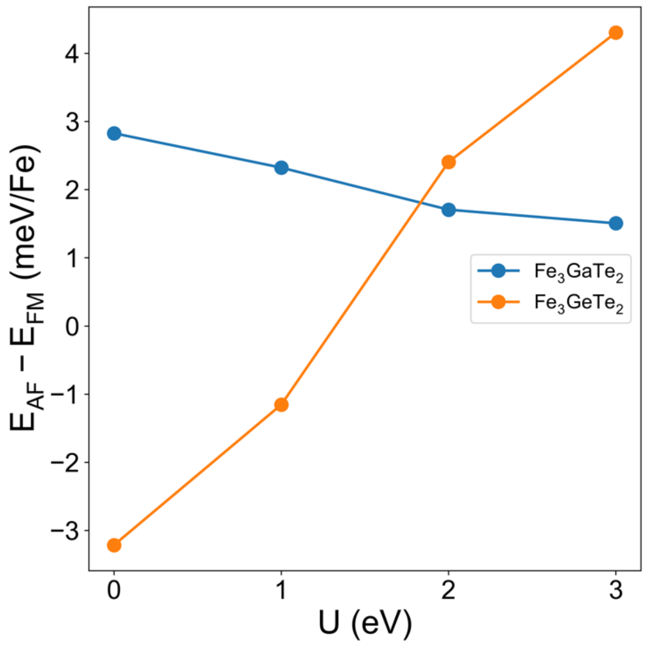

Figure S8. Evolution of the ΔE for Fe_3GaTe_2 and Fe_3GeTe_2 as a function of the value of U. ΔE refers to the energy difference between the AF and FM interlayer couplings (ΔE = E_{AF}-E_{FM}).

Figure S8 shows that for Fe_3GaTe_2 the interlayer ground state is FM regardless of the Hubbard U value. On the other hand, for Fe_3GeTe_2 for values of U = 0 and 1 eV there is an AF ground state, while U > 1 captures the FM ground state. However, as previously discussed, the addition of U in Fe_3GeTe_2 results in a substantial reduction of the AF coupling J_{11}, which has a significant impact on the T_C. For such reason, we conclude that besides the interlayer coupling is not accurately determined for Fe_3GeTe_2 at the LDA level, the absence of U correctly captures the inter-plane exchange interactions J_{12}, J_{13} as well the in-plane couplings J_{11} and J_{33}, which are the ones having the strongest effect in the determined T_C.

1.5 Comparison between bulk and monolayer Fe_3GaTe_2

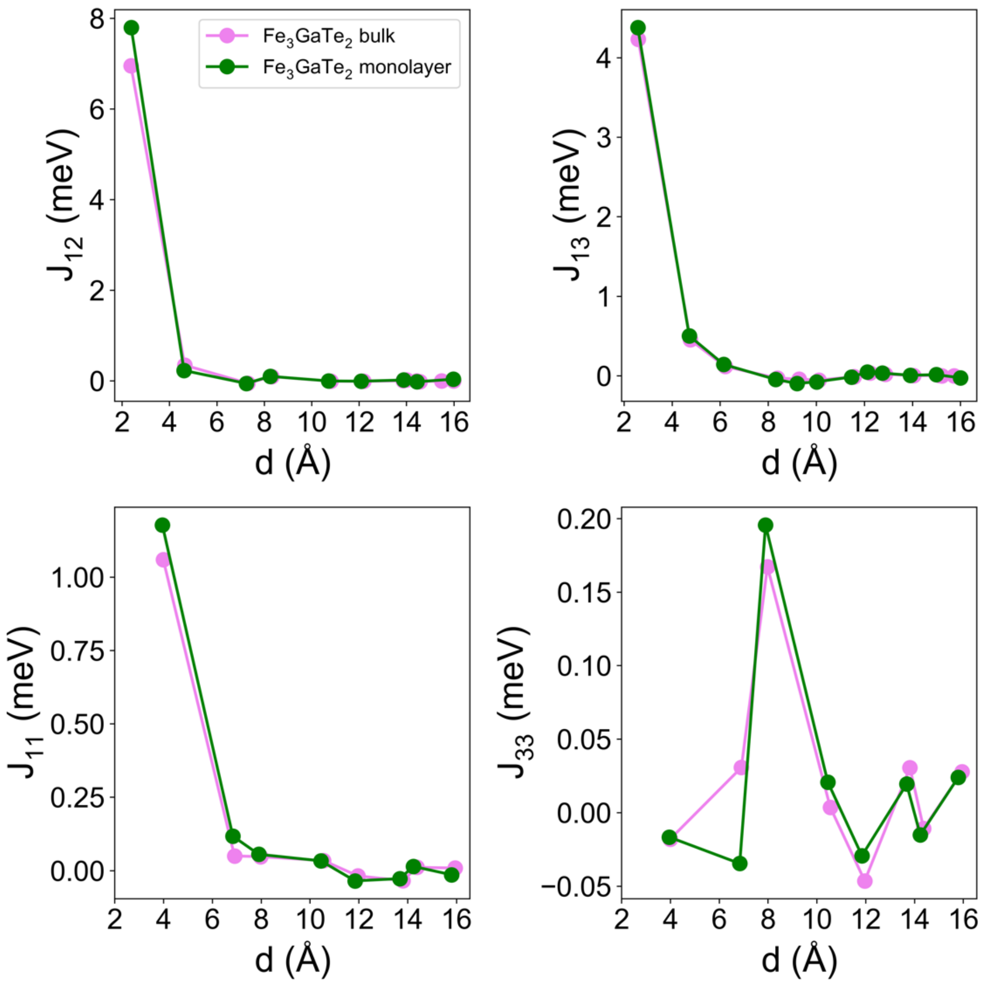

Figure S9. Inter-plane exchange interactions J_{12}, J_{13} (top panel) and in-plane couplings J_{11} and J_{33} (bottom panel) for bulk (pink) and monolayer (green) Fe_3GaTe_2, as well as their evolution with distance.

1.6 Monolayer Fe_3GaTe_2

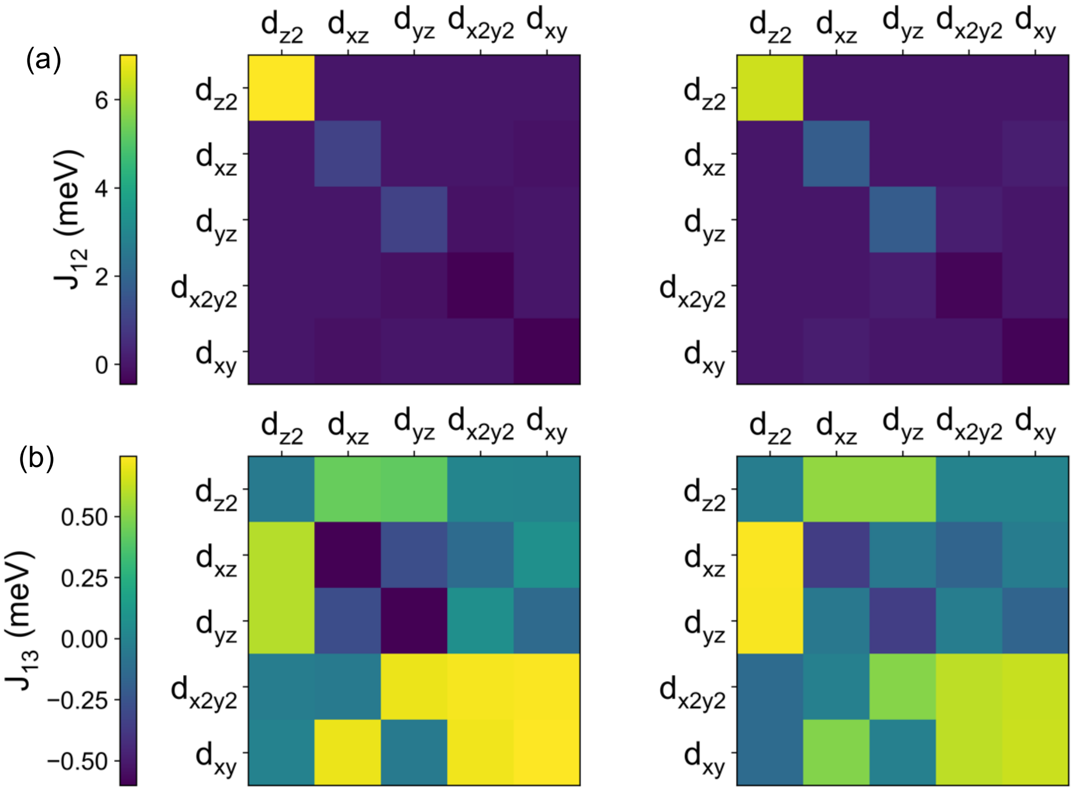

Figure S10. Orbital-resolved inter-plane exchange parameters (a) J_{12} and (b) J_{13} for Fe_3GaTe_2 (left) and Fe_3GeTe_2 (right) monolayers.

We discard the possibility that structural differences between Fe_3GaTe_2 and Fe_3GeTe_2 is the determinant factor to explain the variations in magnetic exchange couplings and T_C. This is confirmed by calculating the exchange couplings J_{12}, J_{13}, J_{11} and J_{33} using the same structure for Fe_3GaTe_2 and Fe_3GeTe_2. As displayed in Figure S11 and Figure 3, we observe that the obtained exchange couplings are independent of the structure employed, and that the differences in T_c between both compounds stem from the distinct contribution of orbitals involved in the stabilization of the long-range magnetic ordering upon substitution of Ga by Ge.

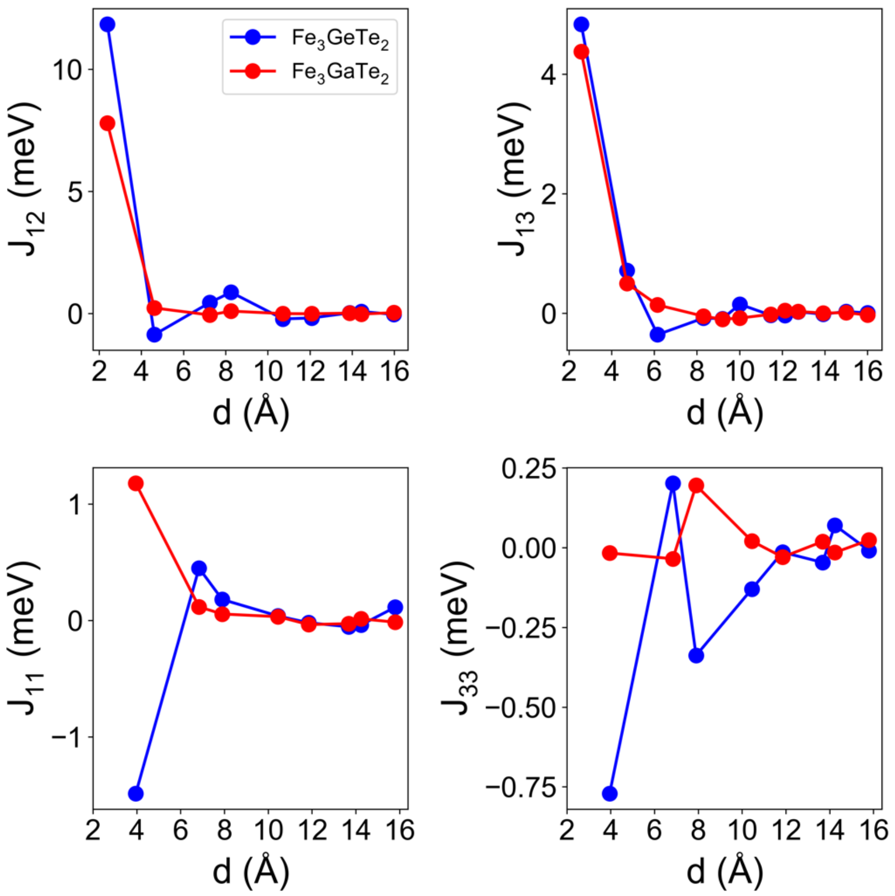

Figure S11. Inter-plane exchange interactions J_{12}, J_{13} (top panel) and in-plane couplings J_{11} and J_{13} (bottom panel) for monolayers Fe_3GaTe_2 (red) and Fe_3GeTe_2 (blue) along with their evolution with distance using the same crystalline structure for both systems.

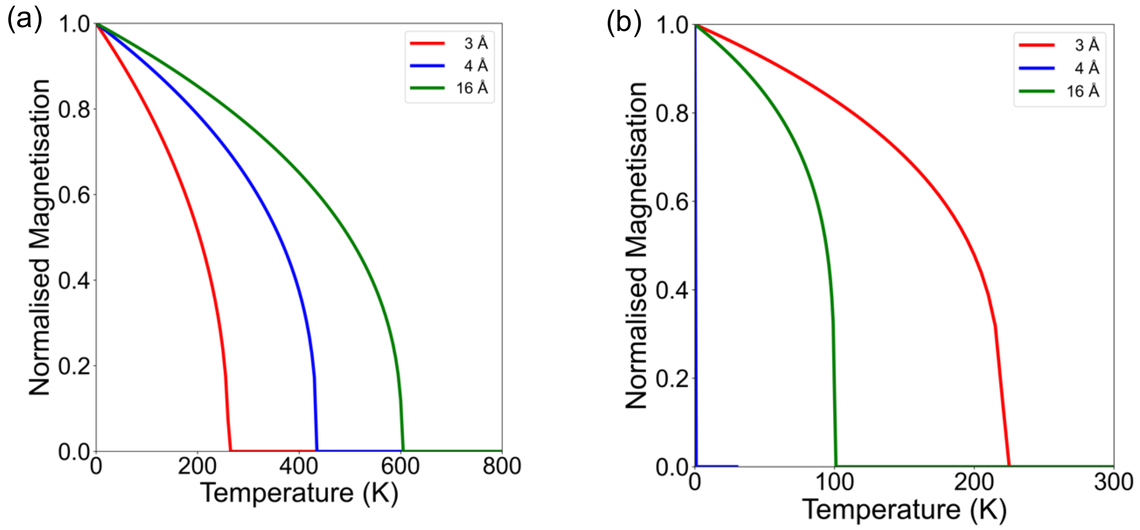

Figure S12. Evolution of the T_C for (a) Fe_3GaTe_2 and (b) Fe_3GeTe_2 monolayers considering interactions up to 3 (red line), 4 (blue line) and 16 Å (green line).

The relevant effect of including J_{11} and J_{33} (interactions up to 4Å) is based on the larger number of J_{11} and J_{33} with respect J_{12} and J_{13} (see Tables S8 and S9). Specifically, besides J_{12} is the strongest FM interaction, there is only one J_{12} coupling per magnetic atom. In contrast, there is a larger number of J_{11} and J_{33} interactions, contributing significantly to the obtained T_C.

Table S8. Values of J_{12}, J_{13}, J_{11} and J_{33} for Fe_3GaTe_2 monolayer, along with the number of nearest neighbours (NN) and the distances between them (d). For the comparison, we include the interactions J_{23} and J_{22}, given that they equivalent to J_{13} and J_{11}, respectively.

Interaction	Value (meV)	NN	d (Å)
J_{12}	7.80	1	2.38
J_{13} (J_{23})	4.36	3 (3)	2.57
J_{11} (J_{22})	1.17	6 (6)	3.95
J_{33}	-0.03	6	3.95

Table S9. Values of J_{12}, J_{13}, J_{11} and J_{33} for Fe_3GeTe_2 monolayer, along with the number of nearest neighbours (NN) and the distances between them (d). For the comparison, we include the interactions J_{23} and J_{22}, given that they equivalent to J_{13} and J_{11}, respectively.

Interaction	Value (meV)	NN	d (Å)
J_{12}	9.87	1	2.41
J_{13} (J_{23})	4.49	3 (3)	2.60
J_{11} (J_{22})	-0.79	6 (6)	3.99
J_{33}	-1.02	6	3.99

The impact of MAE in the T_C for monolayer Fe_3GaTe_2 (Figure S13) is assessed by ranging the MAE from 0 to 8 meV/Fe atom and evaluating the T_c.

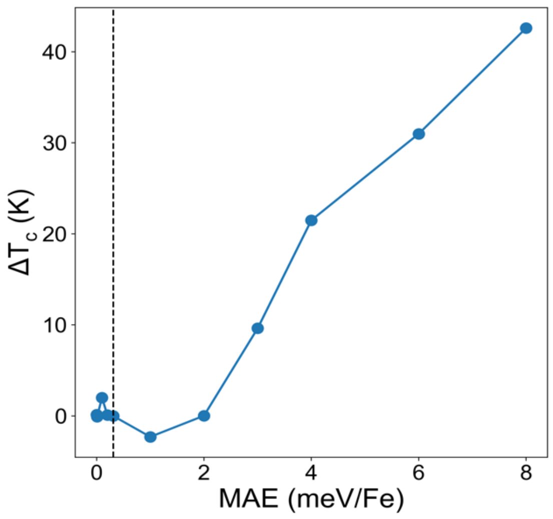

Figure S13. Variations of the critical temperature (ΔT_c) as a function of MAE. Our calculations provide results within an accuracy of $T_C \pm 2K$, which explain the oscillations of T_C for values of $0 < MAE < 2$ meV.

We observe that in the range of MAE < 2 meV, T_C is almost independent of the MAE, which can be understood as a predominant contribution from the isotropic exchange interactions to the T_C. For values of MAE > 2 meV, the Tc steadily increases, reaching to an enhancement of 50 K upon a value of MAE of 8 meV.

2. Strain engineering of Fe_3GaTe_2 monolayer

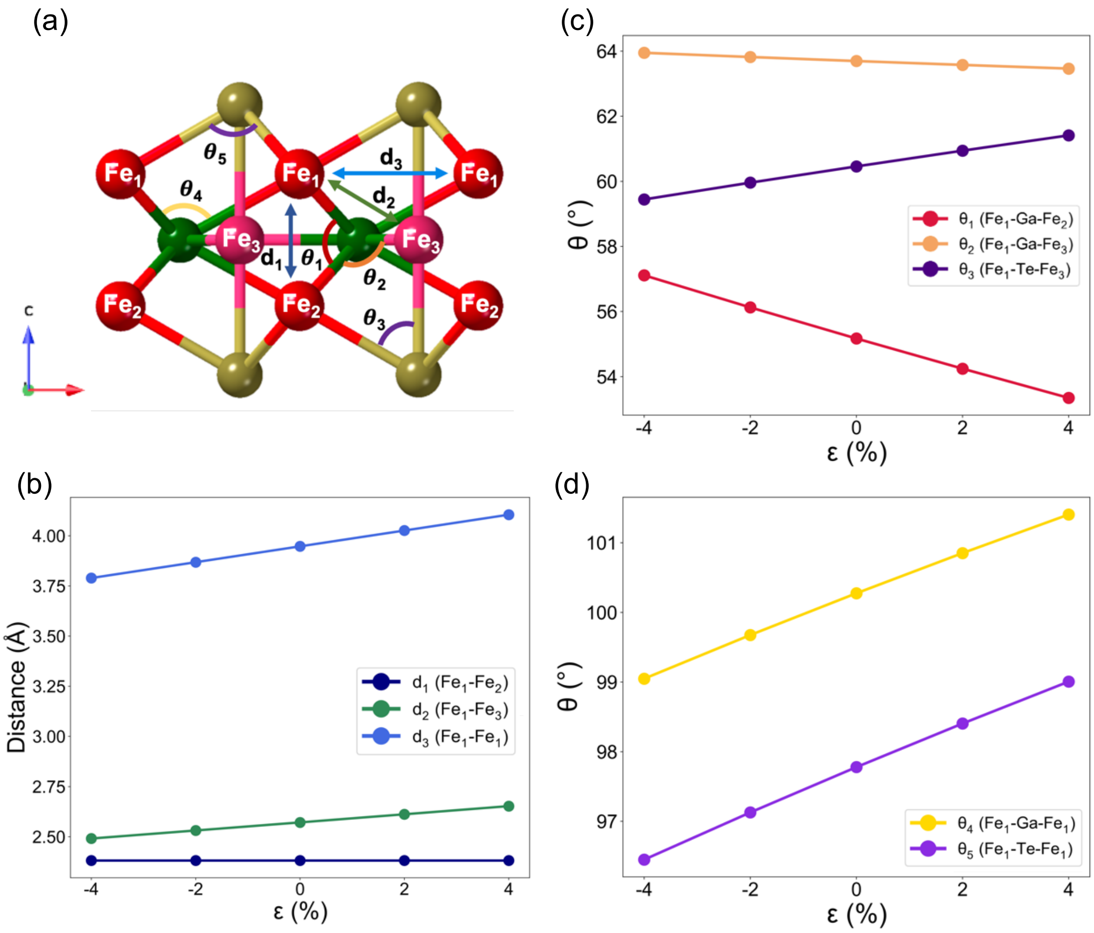

Figure S14. (a) Lateral view of Fe_3GaTe_2 monolayer, labelling the distinct distances (d_1, d_2 and d_3) and angles ($\theta_1, \theta_2, \theta_3, \theta_4$ and θ_5). (b) Evolution of distances (d_1, d_2 and d_3), (c) inter-plane angles (θ_1, θ_2 *and* θ_3) as well in-plane angles (θ_4 *and* θ_5) of Fe_3GaTe_2 monolayer upon applied strain. Note that Fe_1 and Fe_2 are equivalent atoms, therefore each distance or angle involving Fe_1 has its equivalent counterpart within Fe_2. Colour code: $Fe_{1,2}$ (red), Fe_3 (pink), Ga (green) and Te (yellow).

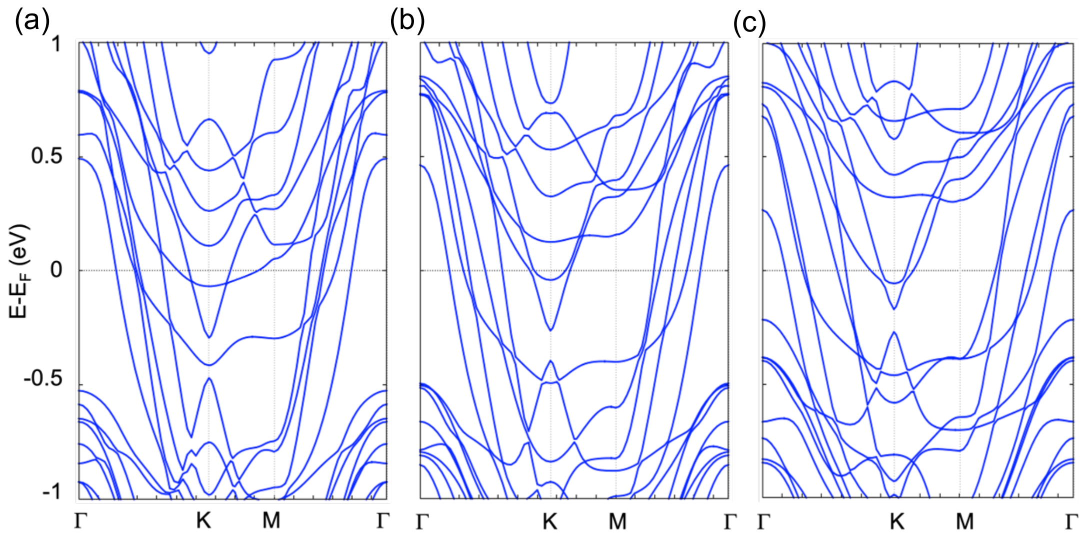

Figure S15. Electronic band structure for (a) -4, (b) 0 and (c) 4% strained Fe_3GaTe_2 monolayer including spin-orbit coupling (SOC) effects.

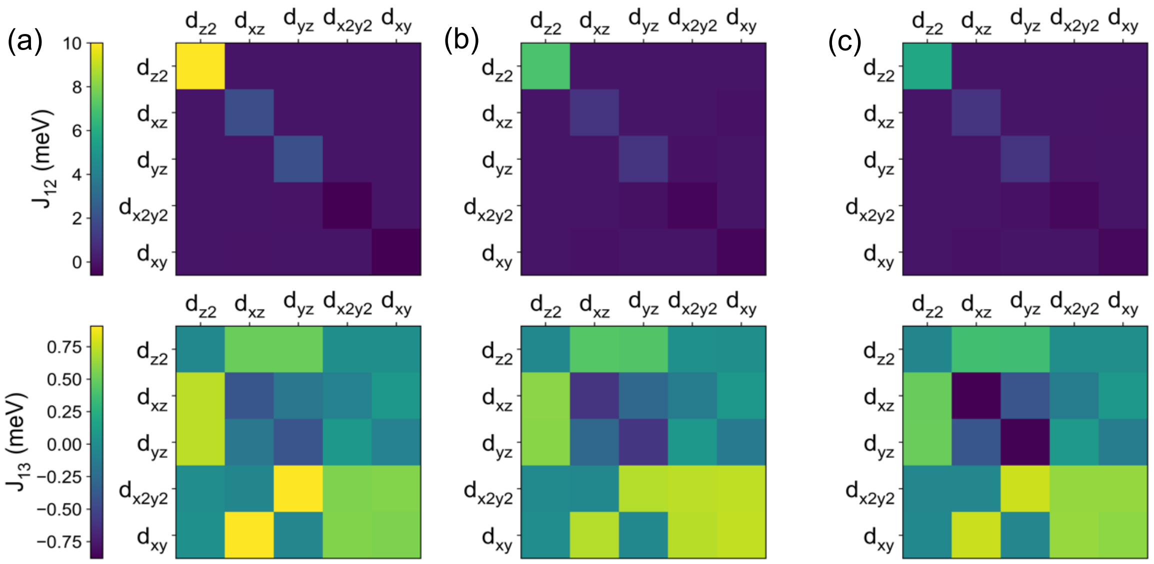

Figure S16. Orbital-resolved inter-plane J_{12} (top panels) and J_{13} (bottom panels) exchange couplings for (a) -4, (b) 0 and (c) +4% strained Fe_3GaTe_2 monolayer.

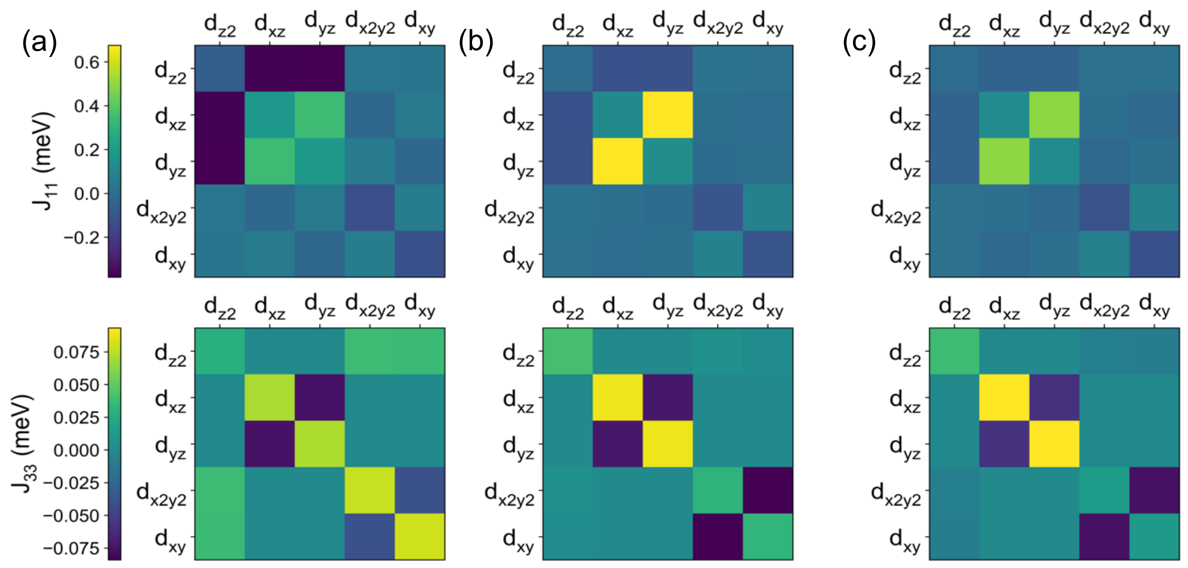

Figure S17. Orbital-resolved in-plane J_{11} (top panels) and J_{33} (bottom panels) exchange couplings for (a) -4, (b) 0 and (c) +4% strained Fe_3GaTe_2 monolayer.

3. Electrostatic doping Fe_3GaTe_2 monolayer

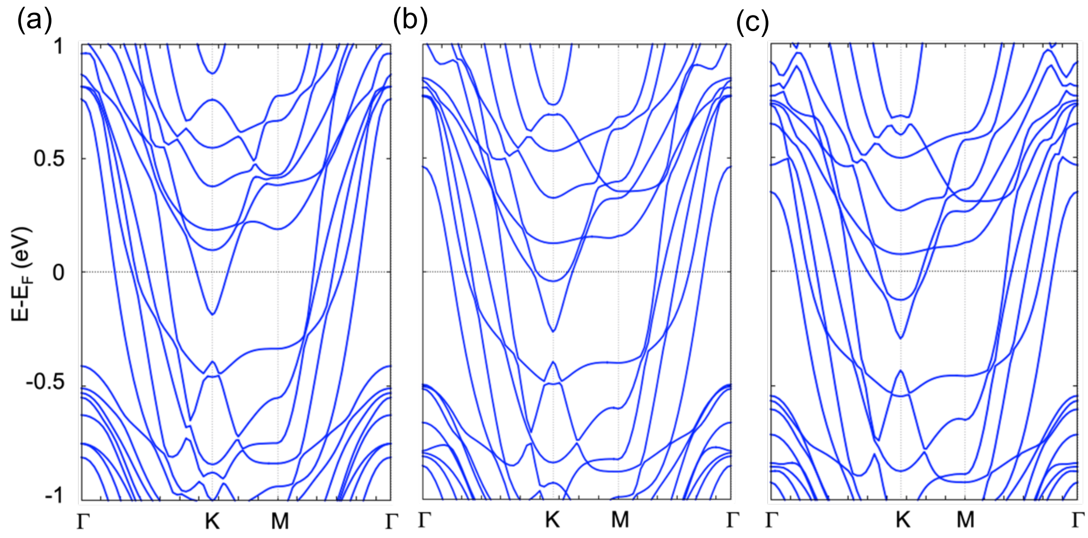

Figure S18. Electronic band structure for doped Fe_3GaTe_2 monolayer with a carrier density of (a) -1.5×10^{14}, (b) 0 and (c) +1.5×10^{14}cm^{-2} monolayer including spin-orbit coupling (SOC) effects. A positive (negative) sign of the carrier density corresponds to an excess of electrons (holes).

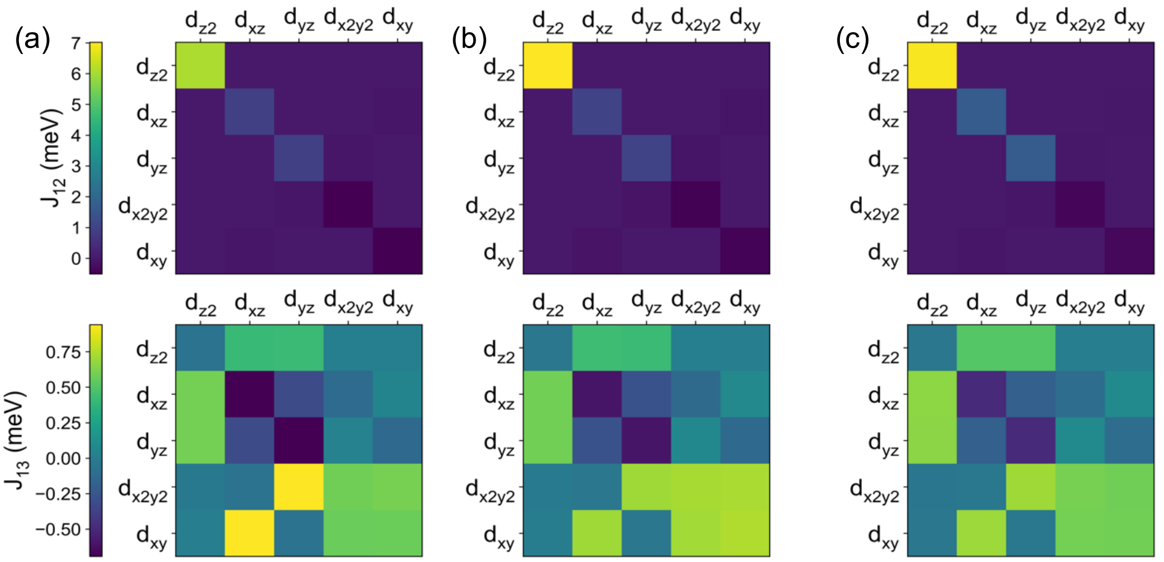

Figure S19. Orbital-resolved inter-plane J_{12} (top panels) and J_{13} (bottom panels) for doped Fe_3GaTe_2 monolayer with a carrier density of (a) -1.5×10^{14}, (b) 0 and (c) $+1.5\times10^{14}$ cm^{-2}. A positive (negative) sign of the carrier density corresponds to an excess of electrons (holes).

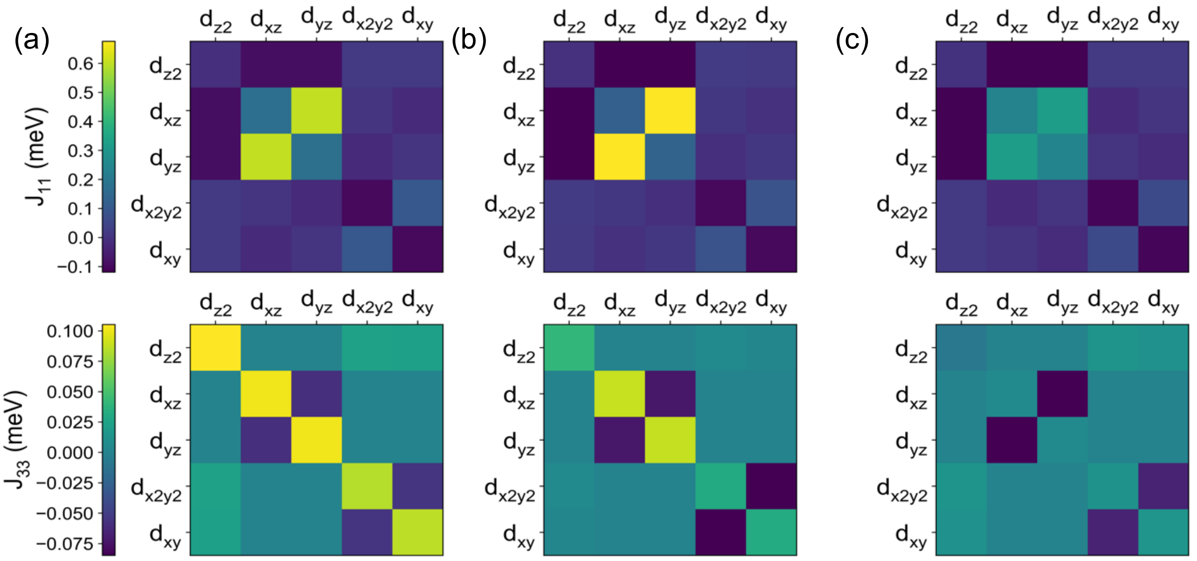

Figure S20. Orbital-resolved in-plane J_{12} (top panels) and J_{13} (bottom panels) for doped Fe_3GaTe_2 monolayer with a carrier density of (a) -1.5×10^{14}, (b) 0 and (c) $+1.5\times10^{14}$ cm^{-2}. A positive (negative) sign of the carrier density corresponds to an excess of electrons (holes).

4. SIESTA calculations

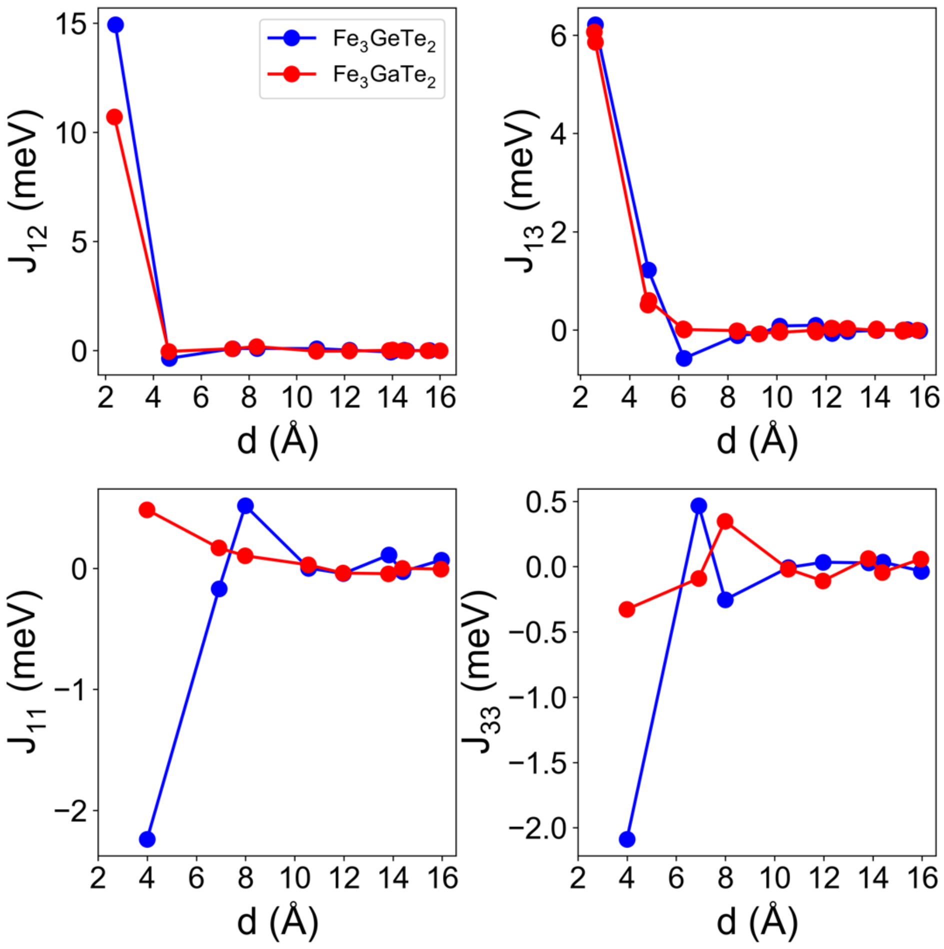

Figure S21. Inter-plane exchange interactions J_{12}, J_{13} (top panel) and in-plane couplings J_{11} and J_{13} (bottom panel) for bulk Fe_3GaTe_2 (red) and Fe_3GeTe_2 (blue) along with their evolution with distance.

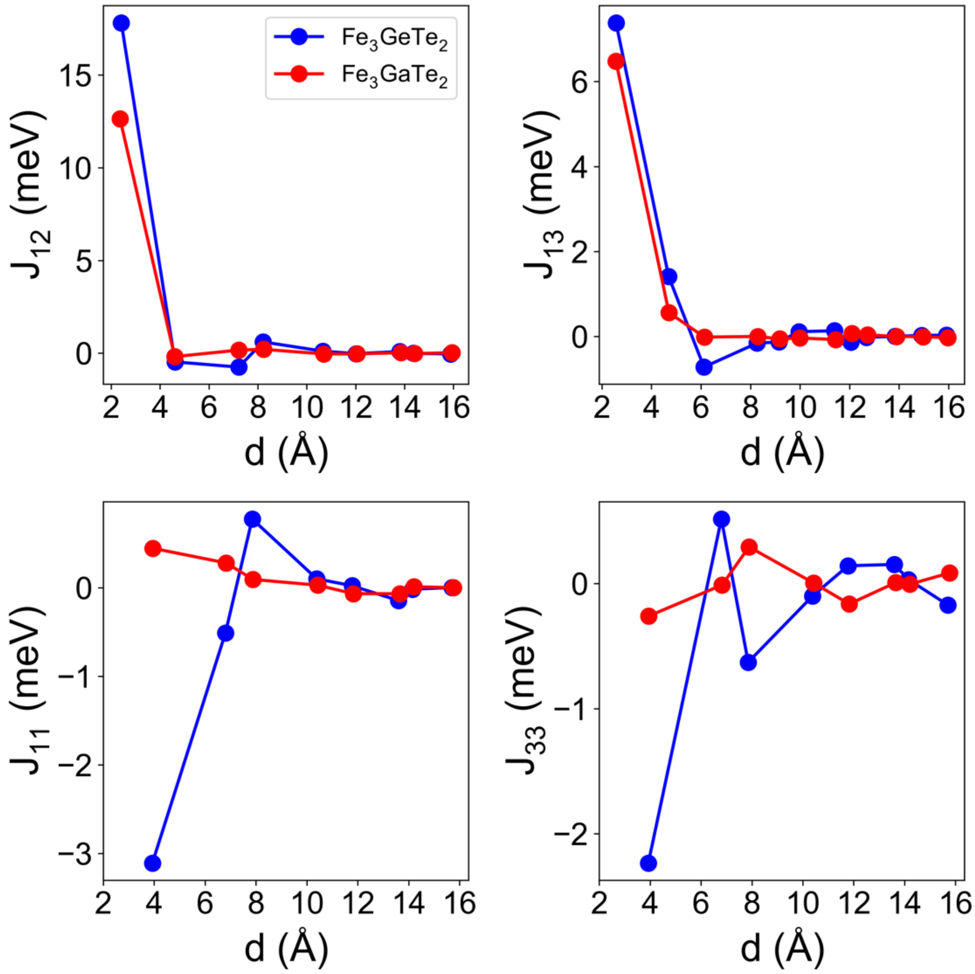

Figure S22. Inter-plane exchange interactions J_{12}, J_{13} (top panel) and in-plane couplings J_{11} and J_{13} (bottom panel) for monolayer Fe_3GaTe_2 (red) and Fe_3GeTe_2 (blue) along with their evolution with distance.

REFERENCES

(1) Chen, B.; Yang, J.; Wang, H.; Imai, M.; Ohta, H.; Michioka, C.; Yoshimura, K.; Fang, M. Magnetic Properties of Layered Itinerant Electron Ferromagnet Fe_3GeTe_2. *J Physical Soc Japan* **2013**, *82* (12), 124711.

(2) May, A. F.; Calder, S.; Cantoni, C.; Cao, H.; McGuire, M. A. Magnetic Structure and Phase Stability of the van Der Waals Bonded Ferromagnet $Fe_{3-x}GeTe_2$. *Phys Rev B* **2016**, *93* (1), 014411.

(3) Deiseroth, H.; Aleksandrov, K.; Reiner, C.; Kienle, L.; Kremer, R. K. Fe_3GeTe_2 and Ni_3GeTe_2 – Two New Layered Transition-Metal Compounds: Crystal Structures, HRTEM Investigations, and Magnetic and Electrical Properties. *Eur J Inorg Chem* **2006**, *2006* (8), 1561–1567.

(4) Zhang, G.; Guo, F.; Wu, H.; Wen, X.; Yang, L.; Jin, W.; Zhang, W.; Chang, H. Above-Room-Temperature Strong Intrinsic Ferromagnetism in 2D van Der Waals Fe_3GaTe_2 with Large Perpendicular Magnetic Anisotropy. *Nat Commun* **2022**, *13* (1), 5067.

(5) Zhuang, H. L.; Kent, P. R. C.; Hennig, R. G. Strong Anisotropy and Magnetostriction in the Two-Dimensional Stoner Ferromagnet Fe_3GeTe_2. *Phys Rev B* **2016**, *93* (13), 134407.

(6) Li, X.; Zhu, M.; Wang, Y.; Zheng, F.; Dong, J.; Zhou, Y.; You, L.; Zhang, J. Tremendous Tunneling Magnetoresistance Effects Based on van Der Waals Room-Temperature Ferromagnet Fe_3GaTe_2 with Highly Spin-Polarized Fermi Surfaces. *Appl Phys Lett* **2023**, *122* (8), 082404.

(7) Shen, Z.-X.; Bo, X.; Cao, K.; Wan, X.; He, L. Magnetic Ground State and Electron-Doping Tuning of Curie Temperature in Fe_3GeTe_2. First-Principles Studies. *Phys Rev B* **2021**, *103* (8), 085102.

(8) Jang, S. W.; Yoon, H.; Jeong, M. Y.; Ryee, S.; Kim, H.-S.; Han, M. J. Origin of Ferromagnetism and the Effect of Doping on Fe_3GeTe_2. *Nanoscale* **2020**, *12* (25), 13501–13506.

(9) Liu, Y.; Stavitski, E.; Attenkofer, K.; Petrovic, C. Anomalous Hall Effect in the van Der Waals Bonded Ferromagnet $Fe_{3-x}GeTe_2$. *Phys Rev B* **2018**, *97* (16), 165415.

(10) Lee, J.-E.; Yan, S.; Oh, S.; Hwang, J.; Denlinger, J. D.; Hwang, C.; Lei, H.; Mo, S.-K.; Park, S. Y.; Ryu, H. Electronic Structure of Above-Room-Temperature van Der Waals Ferromagnet Fe_3GaTe_2. *Nano Lett* **2023**, *23* (24), 11526–11532.
